# An automated sonic tomography system for the inspection of historical masonry walls

**DOI:** 10.12688/openreseurope.15769.1

**Published:** 2023-04-21

**Authors:** Javier Ortega, Marnix F.L. Meersman, Sofía Aparicio, Juan Carlos Liébana, Rodrigo Martín, José Javier Anaya, Margarita González

**Affiliations:** 1 Institute of Physical and Information Technologies (ITEFI), Agencia Estatal Consejo Superior de Investigaciones Cientificas, Madrid, Community of Madrid, 28006, Spain; 2 Faculty of Aerospace Engineering, Technische Universiteit Delft, Delft, South Holland, 2629 HS, The Netherlands

**Keywords:** Sonic tomography, stone masonry, cultural heritage, automation, signal processing, non-destructive testing, photogrammetry, 3D inner reconstruction

## Abstract

**Background:** The conservation of the built masonry heritage requires a comprehensive understanding of its geometrical, structural, and material characteristics. Non-destructive techniques are a preferred approach to survey historical buildings, given the cultural value of their fabric. However, currently available techniques are typically operated manually, consuming much time at operational and processing level and thus hindering their use for the on-site inspection of heritage structures.

**Methods:** A novel automated sonic tomography system was designed and built to inspect and obtain information about the inner structure and damage of historic masonry walls. The system consists of a hitting device mounted on a frame that can be placed adjacent to the wall under analysis. The hitting device can move along the surface within the frame area in X, Y and Z directions, generating the sonic wave. The receiving system is a scanning laser vibrometer, able to measure from the distance the displacement of a focused point over time, recording the wave when it reaches the opposite surface.

**Results: **Six stone masonry walls with different interior geometries were constructed at the laboratory by a professional stonemason. The construction of the walls was carefully documented, including the generation of detailed photogrammetric models of each single stone. The system was applied to survey the six masonry walls. Since the inner morphology of the walls is known, the resulting tomographic images could be compared with the ground truth.

**Conclusions: **Automating the inspection allowed to collect thousands of data in a few hours. New software was also developed to automate the processing of the data. Results are expected to highlight the potential of tomography to obtain quantitative information about the interior of heritage structures, while providing new tools that make the implementation of the technique more practical for professionals. Data, software and models have been made publicly available.

## Plain language summary

When working on conservation projects of historical masonry structures, the main challenge is to ensure its structural safety having little information about the existing building. Uncertainties may concern materials, geometry and existing damage. The success of any structural retrofitting measure, which needs to fulfill the criteria of minimum intervention and compatibility with cultural heritage values, essentially depends on our ability to properly understand the structure. Moreover, the inspection is further limited due to the need of following a non-invasive approach that does not harm the valuable fabric.

The present paper proposes new systems and methods to inspect the interior of historical masonry walls. The system is based on a technique called tomography, which is an imaging technique that can produce images of the cross-section of an object based on the transmission of any kind of penetrating wave. The system designed and fabricated by the research team is meant to obtain images of the interior of historic walls based on acoustic wave propagation. Multiple acoustic waves are generated by means of hitting the wall surface on multiple points. At the opposite side, a remote sensing laser vibrometer measures vibrations from a distance on different points, recording the acoustic signal when it reaches the opposite surface.

The technique is fully non-destructive. The research has specifically aimed to automate the inspection, which, currently, is typically performed manually, consuming much time at operational and processing level. That is why their use is limited in practice. The research acknowledges that detailed information about the interior of heritage structures obtained (inner geometry or damage) is essential for conservation purposes and for making decisions on how to intervene. The system has been tested in six masonry walls at the laboratory and shows that the acoustic wave propagation is clearly influenced by the geometric characteristics of the cross-section.

## Introduction

The built masonry heritage is an outstanding tangible record of technical skills and traditional building technology of its time. Its conservation entails challenging tasks, such as a comprehensive understanding of the geometrical, structural, material characteristics of the asset [ Roca *et al*., 2019]. However, the characterization of such aspects is particularly complex in historical buildings, from which technical plans and material information are hardly ever available. Nevertheless, an increased level of knowledge of the structural characteristics is essential, as it aids the decision-making process on possible interventions that comply with current intervention principles, such as minimum intervention, compatibility and durability, among others.

Inspection and diagnosis of heritage structures are also particularly challenging due to the need of preserving the historical fabric and avoid inflicting further damage. Therefore, the use of non-destructive techniques (NDT) is the preferred approach to inspect historical masonry structures [ ISCARSAH, 2003]. Nevertheless, the general understanding is that the use of non-destructive prospecting techniques for highly heterogeneous materials is mostly helpful to assess existing masonry structures in a qualitative way [ Autiero *et al*., 2021; Binda *et al*., 2000; Schuller, 2003; Valluzzi *et al*., 2018]. In this regard, the most common applications of NDT for the qualitative assessment of masonry structures are: (1) evaluation of the inner morphology and structural layout [ Luchin *et al*., 2018; Riveiro *et al*., 2013; Zielinska & Rucka, 2018]; (2) evaluation of the level of deterioration and damage [ Masini *et al*., 2010; Ramos *et al*., 2018]; (3) quality control and assessment of the efficiency of repair actions [ Binda *et al*., 2013; Valluzzi *et al*., 2018]; and (4) preliminary estimation of elastic mechanical properties [ Martini *et al*., 2018; Miranda *et al*., 2016; Van Eldere *et al*., 2019].

However, in professional practice, the application of NDT in masonry structures is still limited. The majority of the techniques are imported from other fields, e.g. geophysics [ Everett, 2013], and/or developed for more homogeneous materials, e.g. steel and concrete, and are not standardized for masonry structures. As a result, professionals in the field of conservation tend to rely more on literature and expert judgment, also due the high costs in terms of time and money of current NDT [ McCann & Forde, 2001]. Also, they typically resort to more invasive, destructive or minor destructive approaches that allow the direct observation of hidden inner characteristics of the masonry elements. Such include borescope or coring, or a quantitative estimation of the material properties via laboratory testing of the extracted specimens or flat-jack testing. All of which are standardized techniques that help professional gain confidence on them and extend their use in practice [ ASTM C1196-20, 2020; ASTM C1197-20e1, 2020].

Tomographic imaging has attracted great attention in medical and industrial applications [ Khairi *et al*., 2019] because it allows mapping the interior of materials and the visualization of the cross-section, extracting information about defects or discontinuities in a non-invasive manner. However, conventional tomographic inspection methods are difficult to implement and interpret in heritage masonry constructions, as well as being highly time consuming. They are typically carried out manually and, given the need of collecting a great amount of data to carry out the tomographic reconstruction, it becomes an important limitation to inspect large components of a building and one of the main reasons why its use is limited in practice. The main limitations are the limited number of measurements that can be carried out, the inability to inspect the entire structure and build a 3D tomography, coupling and positioning errors associated with manual measurements and the time spent in these measures, among others [ Leucci, 2017]. This situation evidences that there is a need for research in the field that provides new tools and standard procedures that allows an on-site quantitative characterization of the masonry cross-section morphology in a more accurate, faster and reliable way. Detailed information about the cross-section can have a significant impact for a variety of professionals in the field of conservation, from archaeologists, e.g., for documenting purposes, to structural engineers, given that the masonry inner morphology has a decisive influence on the structural behavior of historic masonry structures [ Szabó *et al*., 2023].

With the motivation of overcoming these common limitations that hinder the use of NDT for the on-site inspection of heritage masonry structures, the present research proposes the automation and robotization of acoustic-based inspections, which are fully non-invasive, to perform sonic tomography of masonry walls and provides quantitative detailed information of their interior. The automation of inspection procedures has attracted an important deal of attention in recent years in the field of transport infrastructure [ Zhang *et al*., 2022], but is still an emerging field in the context of cultural heritage [ Carbone, 2018; Ippolito, 2017; Ottaviano *et al*., 2018]. Recent works highlight the potential of service robots and drone systems equipped with sensors and instrumentation for applications in cultural heritage [ Ceccarelli *et al*., 2018], but their application is still mostly limited to 3D surveying and visual inspection [ Bevilacqua *et al*., 2018; Ottaviano & Rea, 2019; Rea *et al*., 2017].

The present work shows a sonic tomography system that automatizes the process by developing a robotic hitting system that generates the acoustic waves. The automation of acoustic inspection has been a recent subject of many studies in the field of inspection of concrete infrastructures, aiming to detect flaws and assess their structural integrity [ Watanabe *et al*., 2015; Li *et al*., 2017; Feng *et al*., 2021; Hoxha *et al*., 2022]. Automated hitting devices have been developed to detect detachment of tiled surfaces [ Caliano *et al*., 2021]. Moreover, the possibility of automatize the inspection of infrastructure using Unmanned Aerial Vehicles (UAVs) has also been a subject of recent research, either by exploring their capabilities for constant inspection tasks [ González-deSantos *et al*., 2019] or by mounting hitting systems to the UAVs for hammering inspections [ Ichikawa *et al*., 2017; Nishimura *et al*., 2022]. An automated ultrasonic tomography system was also developed by the research team [ Aparicio *et al*., 2022], which allowed them to perform numerous tomographic slices along the height of several masonry columns in the Convent of Carmo, in Lisbon (Portugal). The inspection time required to perform a tomographic inspection of one slice was highly reduced to approximately 5 min, collecting a great amount of data per day and building 3D tomographic images of entire columns that provided information about internal cracks and their overall degradation state.

At the same time, there is a need to automate the acquisition system and provide tools that are able to focus on large components of the building (e.g. whole building or walls) instead of partial investigations. For that matter, the present research proposes the use of remote sensing technology, i.e. a Scanning Laser Vibrometer (SLV), which is capable of measuring vibrations (e.g. displacement of points) from the distance, i.e. up to tens of meters. Its application in the conservation of the built heritage has grown substantially in recent years, but is still limited. In structural diagnostics, this technology has been successfully applied for measuring displacements at bridges [ Garg *et al*., 2020; Nassif *et al*., 2005] or ambient vibration testing of cultural heritage [ Diaferio *et al*., 2015; Livitsanos *et al*., 2019; Pallares *et al*., 2021; Wenzel, 2000]. At a smaller scale, they have also been applied for non-destructive diagnosis of artworks [ Castellini *et al*., 2003].

In summary, the paper presents the development of an Automated Hitting System (AHS) for emitting sonic signals, coupled and synchronized with a SLV for the reception of the sonic signal, to perform sonic tomographic inspections of heritage masonry walls. The automated sonic tomography system and its mechanical and electronic components are described in detail in Section 2. Section 3 then presents the methodology used to process the sonic signals and extract the necessary information from the raw captured data, which was also automated to reduce the computational time. The system is applied and tested on stone masonry walls of different geometries constructed at the ITEFI laboratory. The construction of the walls was carefully documented, including the generation of detailed photogrammetric models of each single stone before they were placed. As a result, accurate 3D models of the walls were prepared that include their inner configuration. The results of the tomographic inspections and their comparison with the real inner morphology of the walls is then presented. The paper ends by presenting the main conclusions extracted from the research.

## Methods

### Sonic tomography system

To generate the tomographic images using sound, the transmission method was preferred to pulse-echo because of the easier interpretation of the images. Sonic wave propagation methods are particularly suitable for masonry structures because of the limitation of ultrasonic waves in penetrating heterogeneous materials like masonry, where high frequency waves rapidly attenuate when going through discontinuities [ Maierhofer *et al*., 2003]. Sonic tests involve the introduction of mechanical energy into a solid material, typically using an impact on the surface. The energy propagates through the solid as elastic waves and a receiver located on another point of the solid records the sonic signal, which is typically characterized in terms of velocity or amplitude. The lower frequencies generated by the impact of a hammer or a similar hitting device allow the waves to penetrate deeper into the material even though they offer a lower resolution.

In this work, the sonic wave transmitted between the emitter and the receiver is called a ray, by analogy with X-ray tomography. However, even though the sonic transmission between emitter and receiver will rarely follow a straight line, such assumption allows generating a tomographic image that can still provide valuable information about the interior of the structure. The extraction of the tomographic image requires to emit and receive a large number of sonic rays that cover the whole cross section. The tomographic image and 2D or 3D cross-section image reconstruction can be obtained from the velocity measurements of the sonic pulses transmitted through the specimen. Conventional tomographic equipment consists of multiple transducers placed around the material surface, covering the cross-section under study to obtain a high-quality tomographic image. Following previous experiences from the research group in developing automated tomography systems [ Aparicio *et al*., 2022], the solution adopted for the proposed system is to reduce the number of transducers (emission and reception) by moving them around the material.

**Figure 1.**
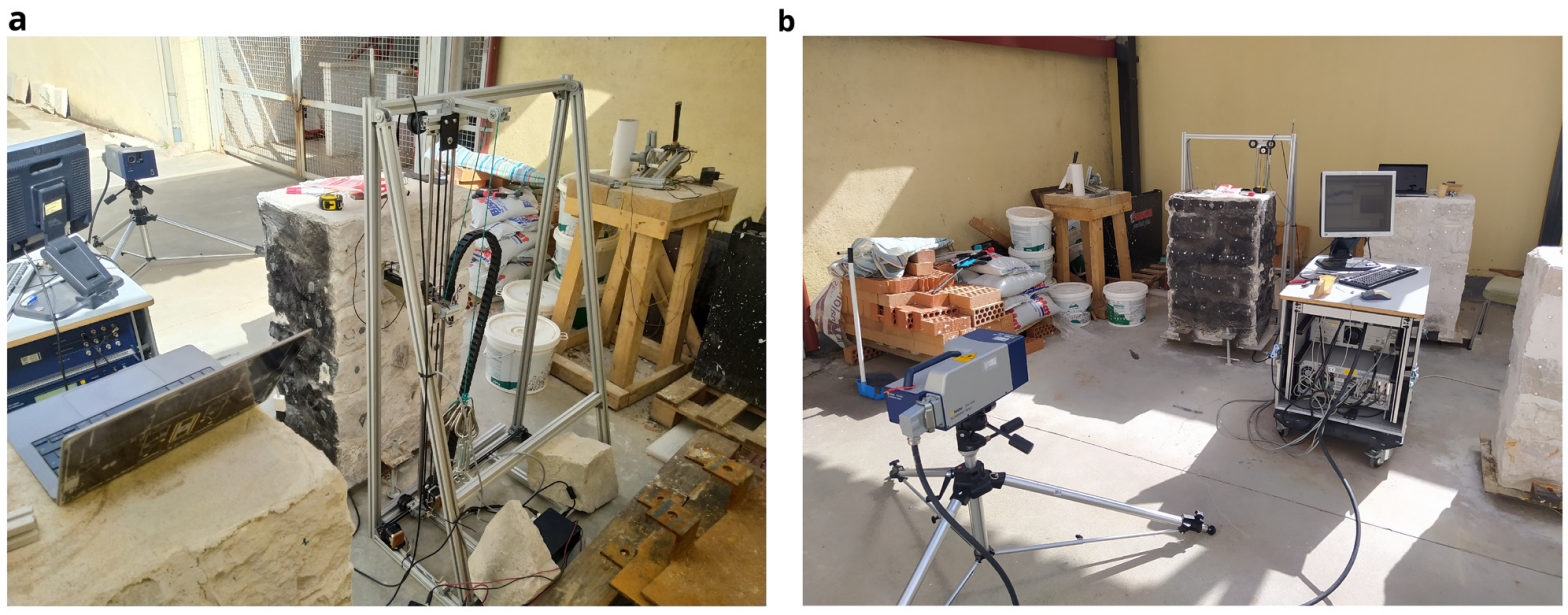
Automated sonic tomography system: ( **a**) AHS for emission; and ( **b**) SLV for acquisition.

Therefore, the new automated sonic tomography system designed and fabricated has two main components: (1) AHS for emission; and (2) SLV for acquisition ( Figure 1). The novel system is expected to provide quantitative information about the inner morphology of masonry structural elements. Even though the system has only been tested in the laboratory, the objective is to provide tools for the on-site inspection of heritage buildings. The main objective of the emission system is to reduce the time consumption at an operational level, improving the applicability of the system on real case studies. For that matter, the AHS consists of a hitting device coupled to an automatic Cartesian robot mounted on a vertical frame. The details of the robot are explained in the next section, but the hitting device essentially consists of a movable metal slug attached to a spring, inserted within a linear solenoid. By controlling the current running through the solenoid, the slug can be pulled against the spring and then released, being pushed forward towards the surface of the wall and generating the sonic wave. The hitting device is able to move in X,Y and Z within the frame. The frame can thus be place close to the wall surface under analysis and the hitting device can move along the surface and hit it on predefined positions. An electronic system was developed to control the robot, including: (a) the movement of the hitting device along the test area; (b) the positioning of the hitting device on the surface; and (c) the hitting, namely velocity and number of hits.

The main objective of the acquisition system is to reach and receive the sonic signal at any point of the structure. That is why remote sensing technology was preferred, which allows the use of the system even if the structure has access limitations. The acquisition system chosen is a scanning laser vibrometer, which consists of 3D rotating heads that can point in any direction and is able to provide information about time-history displacements of the points under the focus of the laser with an accuracy under the millimeter at a range of tens of meters, thus recording the sonic signal when it reaches the opposite surface of the wall.

The synchronization between emission and reception of the sonic signal is achieved by placing a piezo-electric film embedded in plastic at the tip of the hitting device, i.e. the metal slug. This piezo-electric vibration sensor measures the impact of the slug when it hits the surface of the wall and triggers the recording of the sonic signal by the SLV. The mechanical system, the electronic system and the synchronization between emission and reception is described with more detail in the following subsections.

### Automatic Cartesian robot

**Figure 2.**
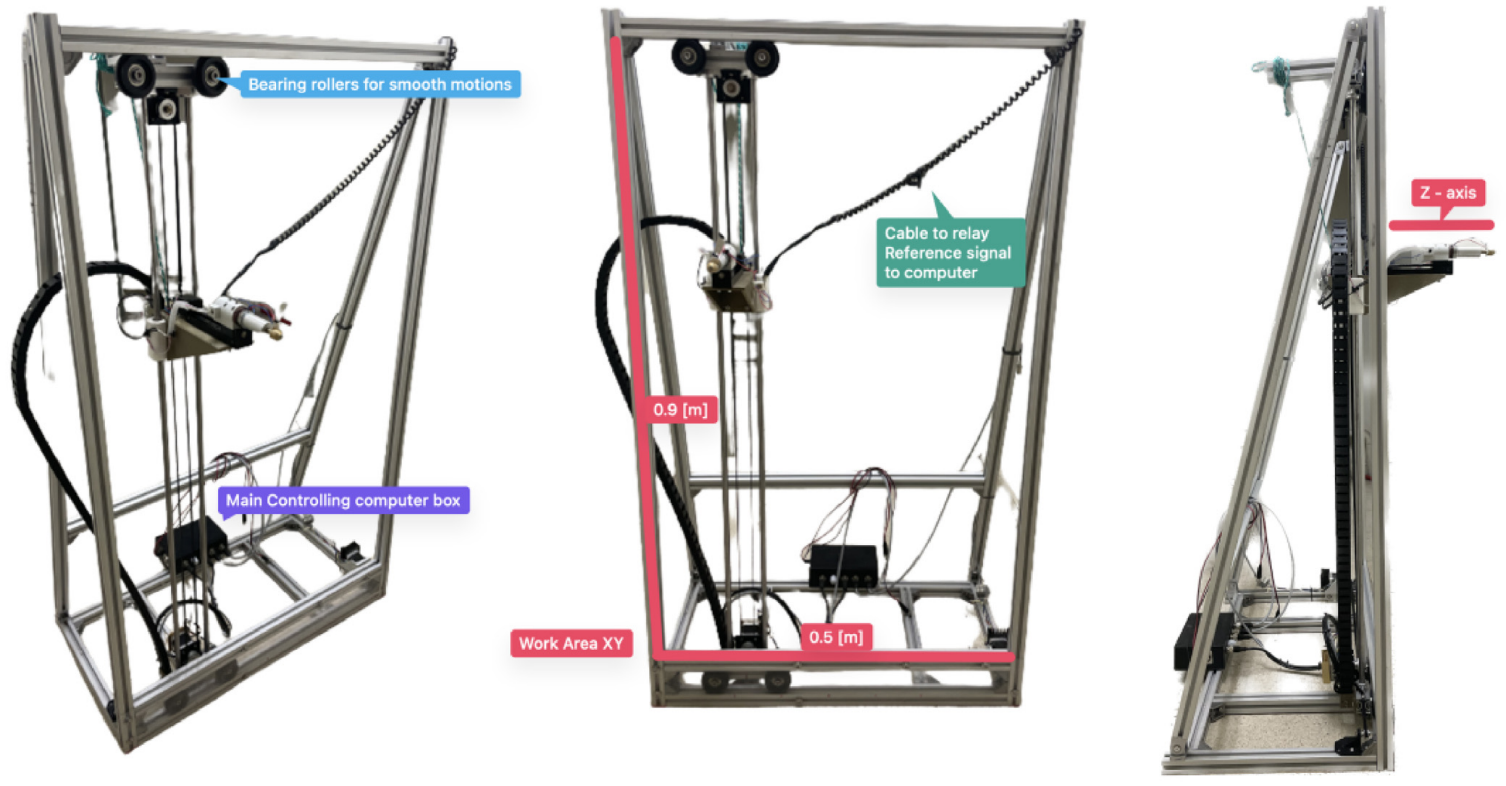
AHS developed, highlighting the main parts and dimensions.

The main role of the mechanical system is to support and guide the movement of the hitting device within a working area. The system designed consists of a vertical frame that define the working plane (XY) of 0.9 x 0.5 m ( Figure 2). A vertical axis consisting of two vertical bars is inserted within the frame supported by two sets of bearing rollers inserted within the top and bottom bar of the frame. A stepper motor controls the movement of the vertical axis through rollers in the X direction. A cart is inserted in the vertical channel and another stepper motor placed at the bottom of the axis controls the movement of the cart in the Y direction.

The hitting device is attached to the cart and the system can move it to any position in the X and Y direction within the working area defined by the frame. The device consists of a metallic head screwed to the tip of the slug within a solenoid. A cap with an internal spring is attached to the metal core of the solenoid. By running current through the solenoid, a magnetic field is created that pulls the slug and attached metallic head (armed state), pushing it against the internal spring. By restricting the current flow, the magnetic field disappear instantly allowing the spring to push the tool head forward at moderate velocity. Once this tool head hits the surface of the wall, it transmits a sonic wave through the wall. To get the AHS close enough to the wall so that the hit reaches the surface, a third stepper motor is also attached to the cart, which moves the hitting device in the Z axis ( Figure 2).

**Figure 3.**
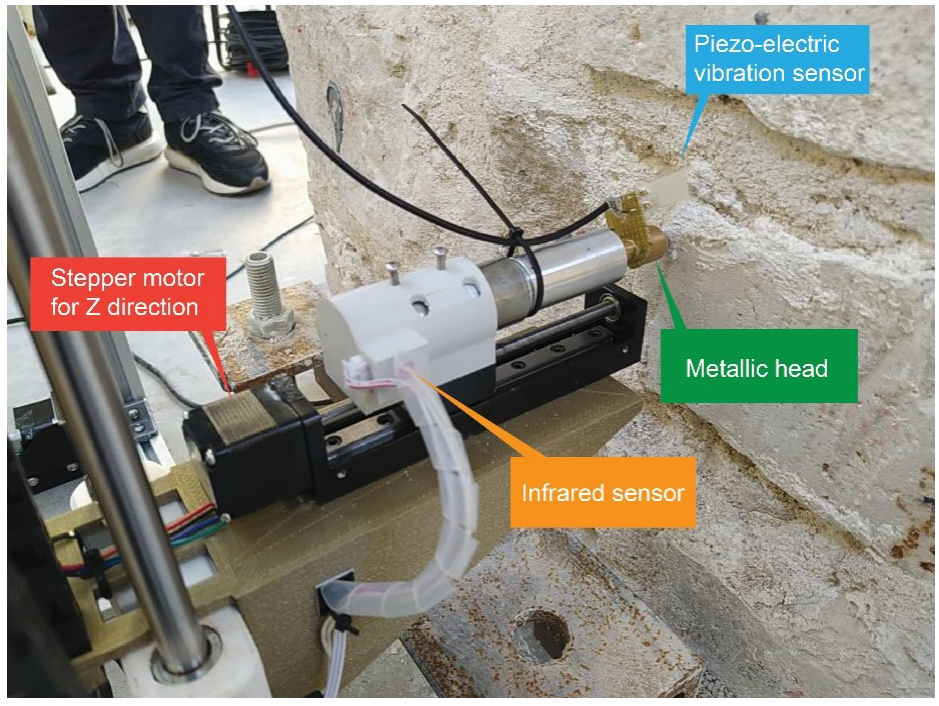
Close-up to the hitting device, highlighting the main parts.

The movement of the hitting device in the Z direction, perpendicular to the wall surface, was essential to adapt it to the irregular surface of common masonry walls. The surface of such walls is rarely planar. For example, some of the walls inspected during the present research had protruding and recessing stones with depth differences of several centimeters along the same wall surface ( Figure 3). The stepper motor is thus able to move the hitting device 15 cm in the Z direction adapting it to the irregularity of the surface. To detect the proximity to the wall surface, an infrared barrier sensor is placed at the back of the hitting device and a pin is attached to the back of the slug ( Figure 3). When the hitting device is moved towards the surface and makes contact, it pushes the slug back, activating the infrared sensor that stops the movement. The motor then moves the hitting device back a few millimeters to position it at the appropriate distance to start hitting.

The last important part of the hitting device is the installation of a piezo-electric vibration or impact sensor on the metallic head of the metal slug. Such sensor is a piezo-electric crystal embedded in plastic that generates a voltage difference when bent. This sensor is able to clearly detect the moment of impact of the slug on the wall, which produces crucial information to synchronize the AHS with the SLV, as will be discussed later.

### The electronic system

The electronic architecture of the robot mainly consists of a serial communication between a PC and an Arduino microcontroller embedded into the robot, i.e. the simple receiver and interpreter of the commands that control the robot. The firmware that runs on the microcontroller is an open-source firmware developed for Computer Numerical Control (CNC) machines named GRBL. In making and interpreting commands, firmware is a low-level class of computer software that provides basic control for a device’s specific hardware, i.e. the hardware of the robot. The firmware only requires the specific dimensions of the robot and its geometric correlations. The microcontroller is responsible for the motion of the hitting device within the frame, actuating on the three motors corresponding to the three degrees of freedom of the robot, which are completely independent of each other. The Arduino board then functions as a mapping between a comment and sending out and receiving digital signals to a variety of motors and sensors, which are listed in Table 1.

**Table 1.** Summary of electronic hardware of the AHS with their respective function.

Component	Function type	Description
2x NEMA 17 stepper motor	Motion	Microstep motor controlling X and Y motion
1x Linear stepper motor	Motion	Microstep motor controlling Z motion
1x Infrared limit switch	Emission	High precision probe to detect contact with the wall surface
1x 24V linear solenoid	Emission	Actuation of the hitting device at certain frequency
1x Piezo-electric vibration sensor	Emission	Determining the moment of impact of the hitting device
1x Arduino Uno 3	Controller	Microcontroller with GRBL firmware
1x Arduino CNC Shield	Controller	Arduino pin remapping board for easy connection with external electronics
3x Stepper motor drivers	Controller	Current limiters transforming the current of the Arduino board to the specific needs per stepper motor

Customized control software with GUI (Graphical User Interface) was developed to control the robot, tailored to the specific needs to perform tomographic surveys on stone masonry walls. For example, the control software was required to generate an evenly space grid over the work area and record the precise locations where the hit took place. At the same time, given the irregularity of the wall surface, the selection of the points in the grid needed to be flexible, allowing easy micro adjusting in real time. Efficiency and speed, while maintaining accuracy were the main design objectives of the control system. Tomographic surveys demand a high number of survey points and a high number of hits per emission point and reception point. Thus, the optimization of the motion has an important impact on the final duration of the full survey performed on a wall.

**Figure 4.**
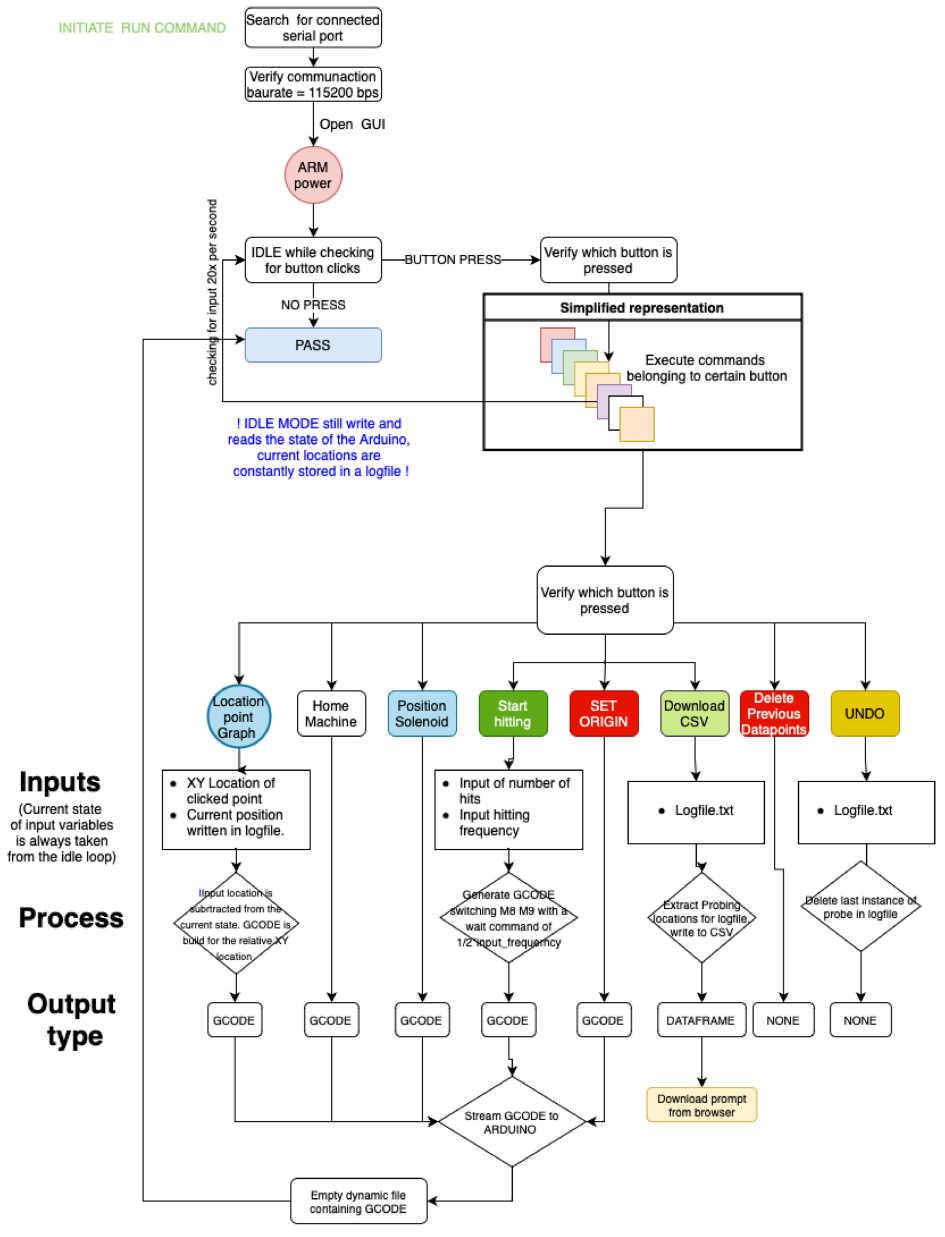
Simplified control software diagram.

***Back-end software.*** A schematic overview of the control software is shown in Figure 4. The software that communicates with all the hardware attached to the main computer was developed in Python using a graphical user interface. The program first checks for user inputs. Once one is detected, it executes the standard commands that are assigned to that user input. Most inputs translate into the physical motion of the robot. However, the software must also allow the exportation and manipulation of the data recorded by the robot.

Python is used to process the commands input in the GUI and reformat them into G-Code (Geometric Code) that can be read and executed by the Arduino microcontroller to control the motion of the robot. The commands executed by the robot include: (i) turn solenoid on and off; (ii) position hitting device at the wall surface, i.e. move hitting device forward (Z direction) until trigger of the infrared sensor; (iii) bring hitting device to retracted position, move hitting device back in Z direction; (iv) move hitting device in X and Y direction at a specific speed; and (v) emit sonic waves, i.e. hit wall surface, at a specific frequency.

Apart from G-Code related actions, the software also has to allow the user to define a survey mesh over the wall surface. By taking in the number of survey locations in the vertical and horizontal direction, the software generates a grid of coordinates that can be adjusted manually by the user. Lastly, it is important to not only generate the grid over the wall surface but also to record all the motions in time, i.e. which grid point was tested first and whether any micro-adjustments were made to the original grid space. For that purpose, all data is recorded automatically in a log file and can be exported as a simple excel file.

**Figure 5.**
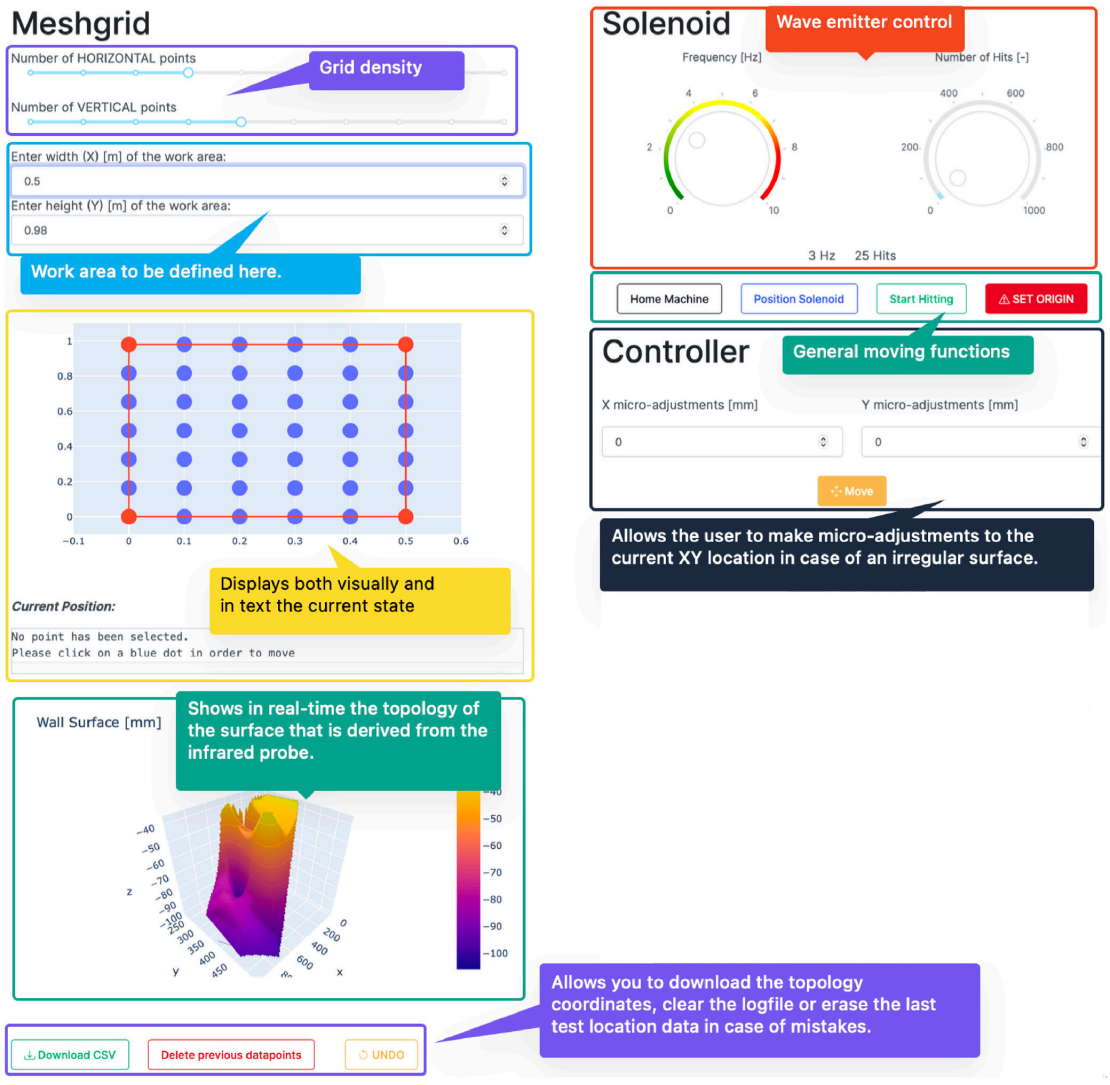
User interface designed to control the robot.

***Front-end software and controlling GUI.*** Figure 5 shows the user interface designed to control the robot with explanations of the different sections. The upper left section (Meshgrid) is where the grid space can be generated and controlled using sliders and input regions for defining the working area. Each dot inside the grid space plot can be clicked and the adequate G-CODE command will be generated for the robot to move to that position. The bottom left section updates in real time and shows the topology of the surface (Z dimension) of each point of the grid. This surface mapping can then be overlaid with the wall surface, such that the emission positions can be precisely located.

The upper right section (“Solenoid”) control the emission: (a) definition of the emission frequency, i.e. hits per second; and (b) number of hits per emission point. After hitting the ‘Start Hitting’ button, the emission will start according to these definitions. The other buttons available in this area are: (i) ‘Set Origin’, which establishes the current position of the hitting device (X, Y and Z coordinates) as the origin; (ii) ‘Home Machine’, which moves the hitting device to the home position; and (iii) ‘Position Solenoid’, which positions the hitting device at the wall surface, i.e. move hitting device forward (Z direction) until trigger of the infrared sensor. The last section (Controller) contains the controls to micro-adjust the planar state of the robot, i.e. shifting the x- and y-axis by a few millimeters. The designed GUI and control software are published open source in GitHub and include a tutorial [ Meersman, 2023a].

### Synchronization of emission and reception

The receiving system is a commercial SLV from Polytec (PSV-400), which consists of a 3D rotating head that can point a laser beam on any point of the structure from the distance and measure vibrations. SLVs use the Doppler effect to measure vibrations. By pointing a laser to a certain object, the laser beam will be reflected from the object. The SLV collects the reflected light, which varies over time due to the movement of the object. The characteristic of the motion of the specific point targeted by the laser can be measured from the wavelength variations of the reflected light, which are measured by superimposing the original and reflected signals.

**Figure 6.**
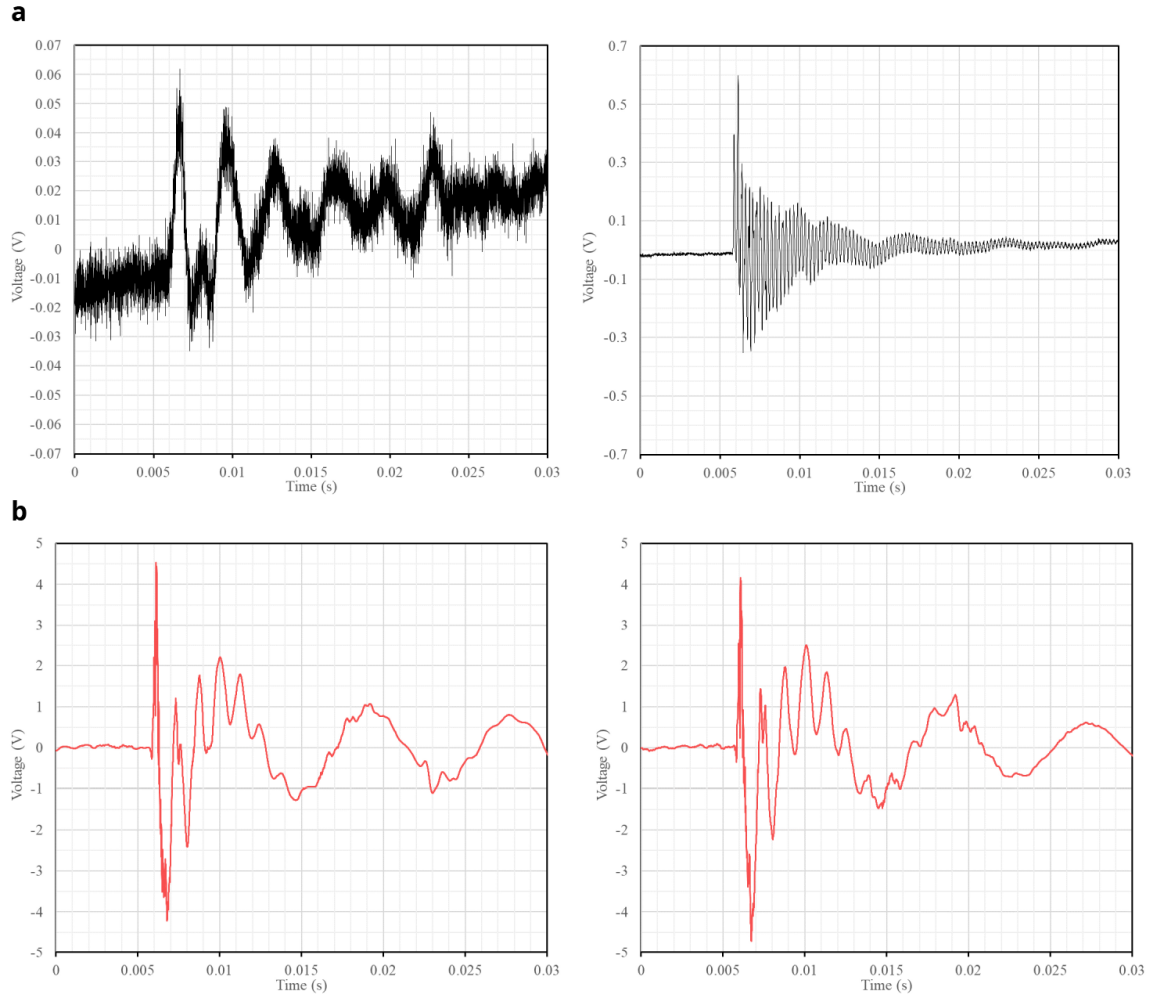
Examples of raw signals recorded when the slug hits the wall surface: ( **a**) vibrations at two different points of a wall measured by the SLV; and ( **b**) respective vibrations captured at the tip of the slug.

The sampling rate of the SLV is set to 256k samples per second, which allows for a sufficient resolution of the receiving signals ( Figure 6). To synchronize the reception with the emission, the piezo-electric vibration sensor shown in Figure 3 is placed at the tip of the hitting device. The sensor measures the vibrations at the tip and clearly detects the precise moment when it hits the wall surface ( Figure 6b). The sensor is connected to the SLV data acquisition system and the setup is configured for the SLV to measure the vibrations at the point targeted by the laser beam when the signal obtained from the vibration sensor exceeds a certain threshold that is clearly associated with the hitting time. Figure 6 shows an example of both the signals measured by the SLV and the vibration sensor. The signal from the vibration sensor is consistent in every hit, which is essential to guarantee a proper synchronization between the emission and reception. Once the same time frame is ensured for both emission and reception, the travel time of the acoustic throughout the media can be measured.

### Processing of the sonic tomography information

As previously mentioned, a big number of measurements taken from different angles is necessary to obtain an appropriate tomographic image that is able to show the cross-section of the element, allowing the detection of cracks or discontinuities (i.e. inner morphology). The need for such large number of sonic rays results in a large quantity of data per inspection. Therefore, there is also a need for automating the processing of the data to obtain the tomographic images of the walls. The methodology to process the data consists of two steps: (1) extraction of the sonic information from raw captured data; and (2) generation of the tomographic image.

### Extraction of the sonic information from raw captured data

**Figure 7.**
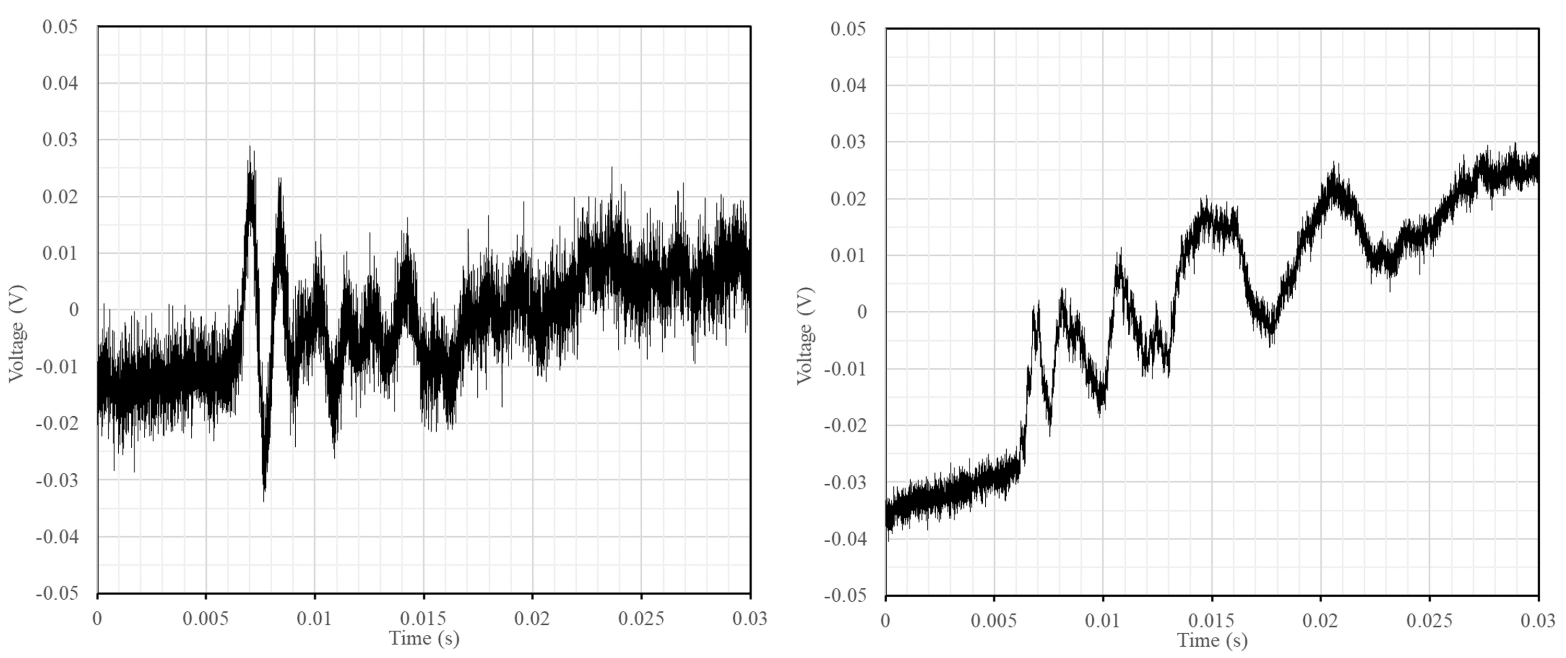
Examples of common raw signals obtained with a high amount of random noise of low and high frequencies.

The proposed system with AHS and SLV allowed to automate the tomographic inspection, greatly reducing the operational time. However, the reception using SLV instead of another device, e.g. a highly sensitive accelerometer, leads to a high amount of random noise over most of the signals ( Figure 7). To extract the sonic wave information that we need to obtain the tomographic image, namely the travel time since we will be using velocity measurements for the tomography, three tasks are necessary: (i) noise elimination from emission and reception signal; (ii) acquisition shifts, compilation and averaging of the signals; and (iii) determination of acoustic wave travel times.

**Figure 8.**
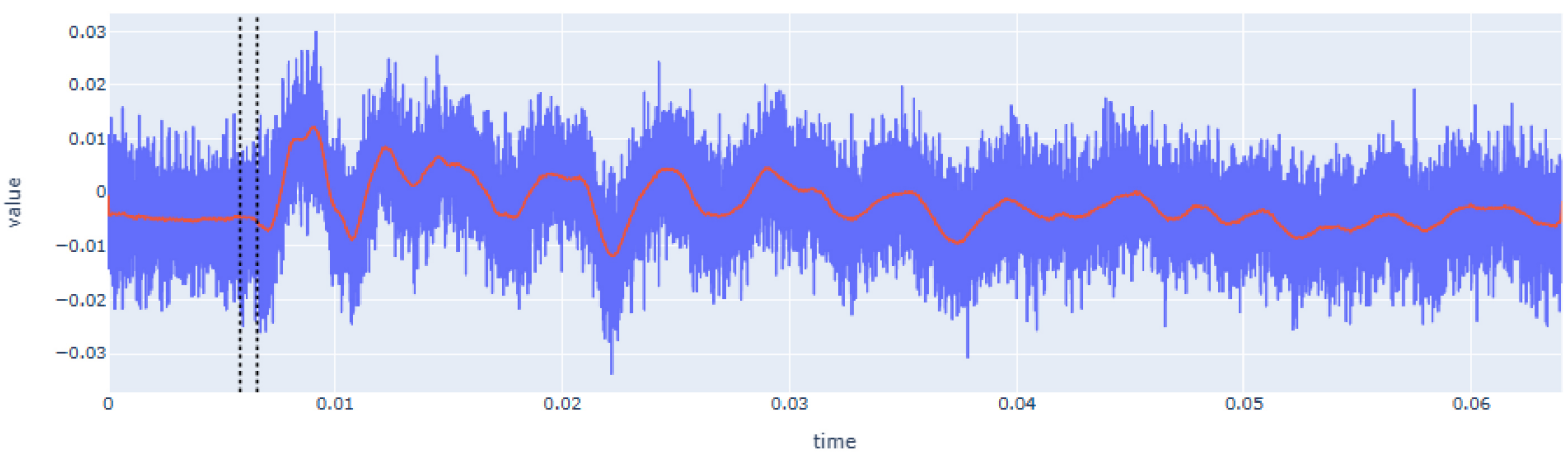
Example of filtered response signal (raw signal in blue and filtered signal in red). X-axis (time) is in seconds (s) and Y-axis (value) is in voltage (V).

The elimination of the noise during the raw data pre-processing was not straightforward. Common filters (e.g. low-pass or smoothing functions) may alter the signal and the identification of the sonic wave front. This has a significant impact on the results, which are based on feature extraction in the time domain. The final method adopted was smoothing the signal by means of convolving the signal in the time domain with a square wave function. Instead of taking the convolution of a square wave function that is as wide as the total width of the waveform, the waveform is convolved with a very small square wave that moves over the complete time series. This process introduces minimal phase shifts that can also easily be accounted for, proving to be a robust filtering method. It also allows the user to iterate this convolution algorithm to get more or less smoothing of the signal. Figure 8 shows an example of a filtered signal compared with the original one.

Computing the travel time, requires processing two waveforms: (a) the emission wave recorded by the vibration sensor ( Figure 6b), which contains the time of emission ( *t _0_*); and (b) the signal received by the SLV ( Figure 6a) which contains the time of emission ( *t _1_*). Since emission and reception are synchronized because they are connected to the same data acquisition system, both signals have the same reference in the time domain. Therefore, the travel time ( *dt*) can be computed as *dt* = *t*_1_ – *t*_0_.

**Figure 9.**
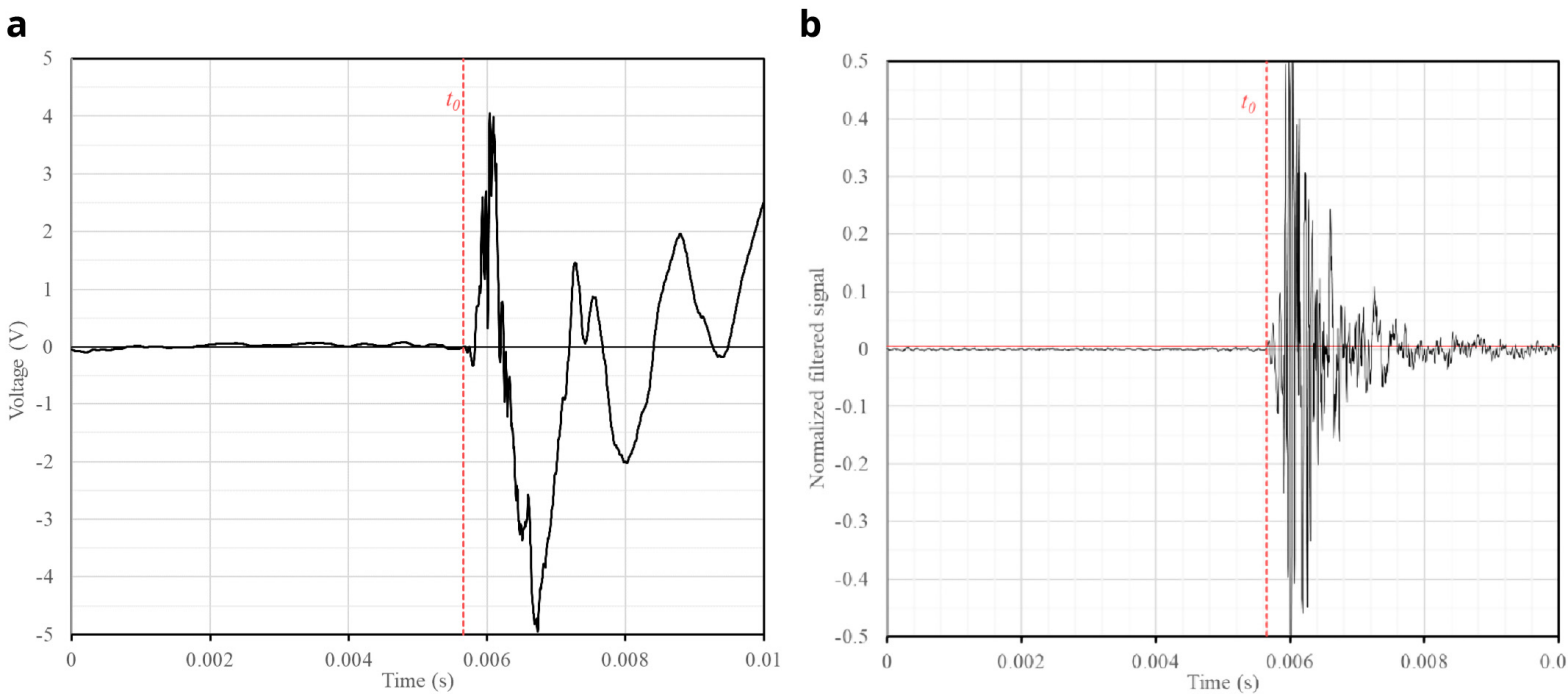
Automatic extraction of emission time ( *t _0_*) from recorded signal of vibration sensor: ( **a**) original; ( **b**) filtered signal.

The signal recorded by the vibration sensor ( Figure 6b) did not contain high-frequency noise, facilitating the identification of the hitting time. Upon further analysis of the time series ( Figure 9a), the peak of the impact signal is always accompanied by a high-frequency vibration superimposed on the signal. These high-frequency vibrations thus are correlated to the moment of impact. With this information, one can apply a high-pass filter to only showcase these higher frequencies ( Figure 9b). After filtering, a simple threshold value criteria was implemented to extract the hitting time, *t _0_*. The threshold value is established as 0.5% of the normalized signal.

The time extraction from the waveform recorded by the SLV was more complex due to the high variability of the signal in terms of shape and noise content. The first step is the noise elimination filter ( Figure 8), which allowed for an easier interpretation and detection of reception time, but further signal processing was necessary to automate the time extraction in a robust way.

The second step was to compile and average all the signals recorded for each sonic ray (i.e. all the signals with the same emission and reception point). To avoid anomalous signals, 10 hits were performed per reception point. The signals with a low signal-to-noise ratio (SNR) were removed and the rest were combined for averaging. However, before averaging they needed to be aligned because, due to a variety of factors (e.g., vibration of the system, variation in the amplitude of the signal, irregularity of the surface, etc.), *t _0_* was not exactly the same in all signals. Therefore, to average the emitting and receiving signals, they must be shifted such that they share the same time domain. After the extraction of *t _0_* for each signal ( Figure 9), the waveforms were aligned, applying the same shift for emission and reception waveforms, since each pair of emitting and receiving signals do share the same time domain. After applying the shift, the subset of signals were averaged, resulting in a single waveform per sonic ray, which also helped further reducing the noise.

**Figure 10.**
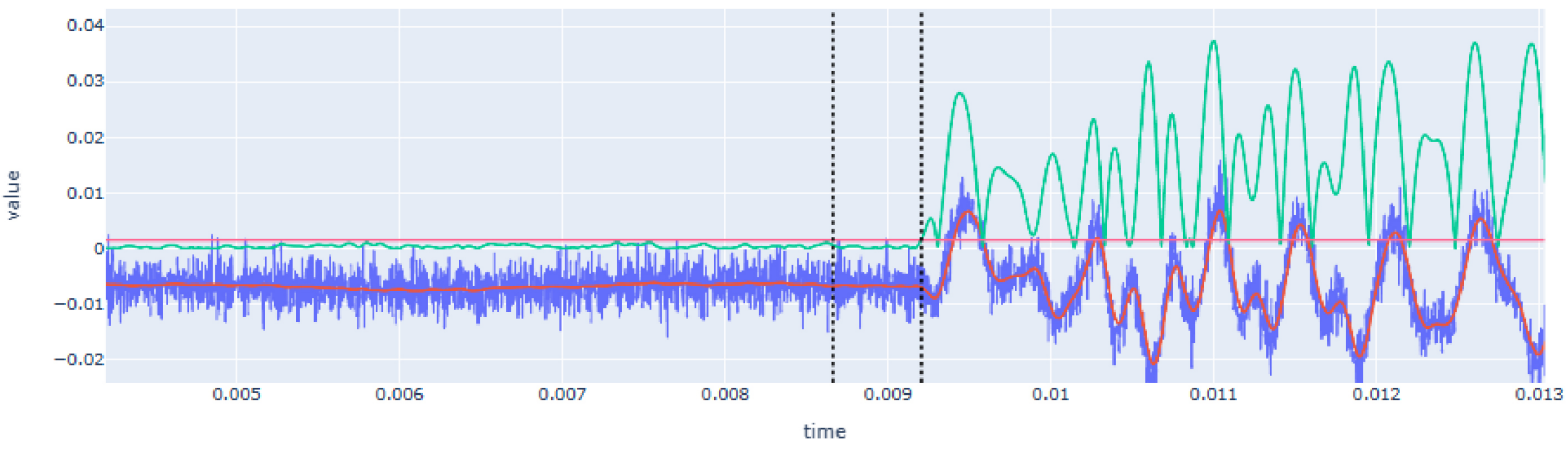
Automatic extraction of reception time ( *t _1_*) from recorded signal of SLV after its transformation using the double rolling aggregate method (green line) and definition of the threshold (red horizontal line). Note that raw signal is drawn in blue and filtered signal in red. The first dotted vertical line corresponds to *t _0_* and the second to *t _1_*. X-axis (time) is in seconds (s) and Y-axis (value) is in voltage (V).

The determination of *t _1_* from the reception waveform was carried out after transforming it with a double rolling aggregate algorithm. This algorithm examines the signal over two sliding time windows side-by-side along the time series and measures changes in the variability of the signal, i.e. by comparing differences of the metrics between the two windows after and before a specific value [ Arundo Analytics, 2019]. Figure 10 shows an example of the transformed signal and the automatic detection of the reception time. Since *t _0_* is already known at this step, the detection algorithm looks for the highest amplitude of the transformed signal before *t _0_*. This value is related to the amount of noise that exists before the hit. This maximum value plus a user-defined percentile increase then becomes the trigger threshold. The first time this threshold value is exceeded by the time series after *t _0_*, becomes the final *t _1_*.

**Figure 11.**
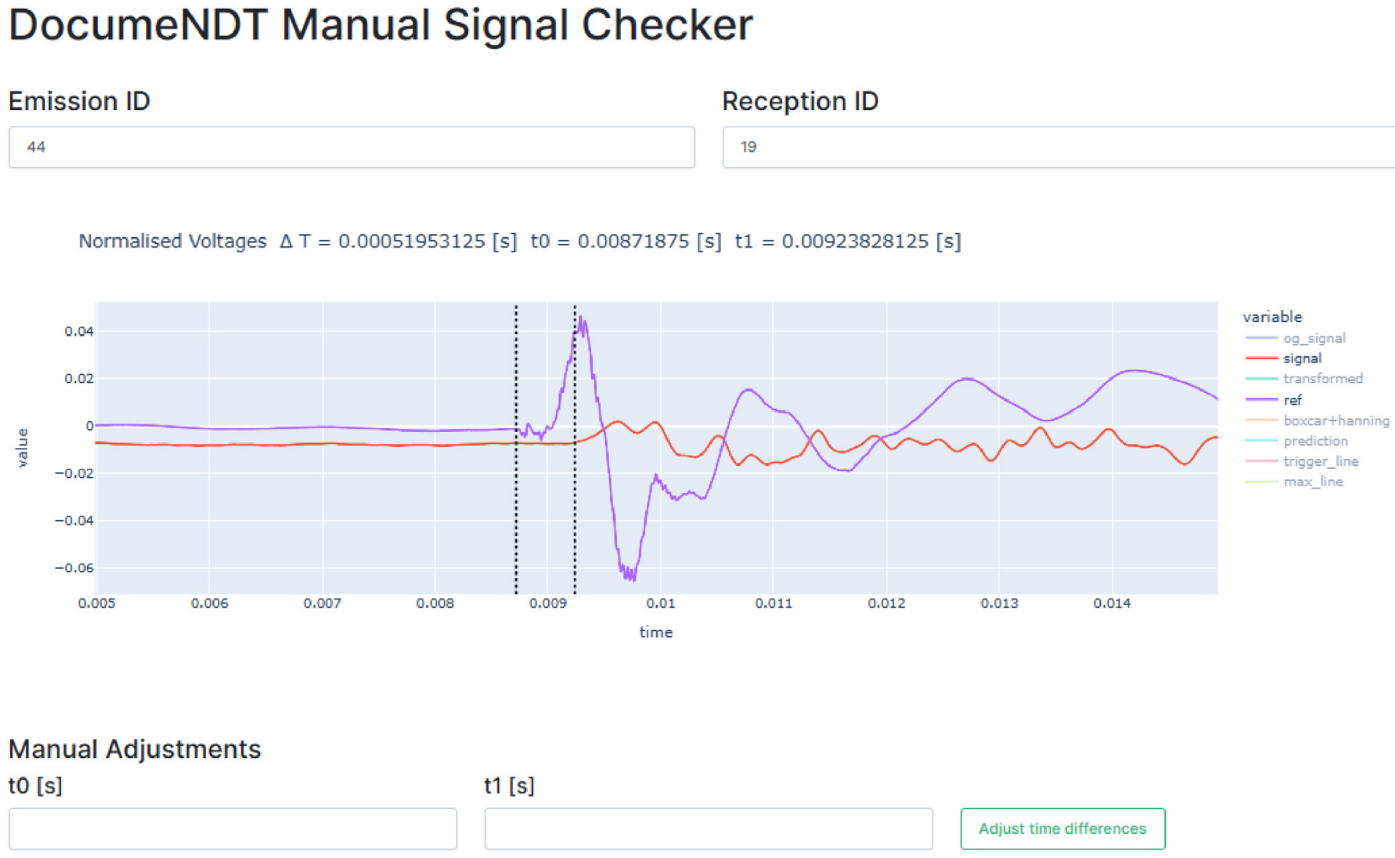
Graphical User Interface to manually verify each signal. The bottom two input fields allow for real-time manual adjustments and the legend at the right of the graph allows to visualize the original and processed signals. X-axis (time) is in seconds (s) and Y-axis (value) is in voltage (V).

The process was repeated for all signals and confirmed its robustness. However, due to the high variability of the signals and the overall high amount of noise recorded, the process should allow to verify all signals manually if some anomalous value is detected. The automatic data processing software is also published open source in GitHub and include a tutorial [ Meersman, 2023b]. The user interface is shown in Figure 11 and, besides showing the automatically detected times and travel time, allows the user to verify all signals and manually enter new values for *t _0_* and *t _1_* that overwrite the output of the automated algorithm. All signals are loaded and the user is able to select which signals to manually check. Additionally, the graph includes an interactive legend (on the right side) that allows to visualize or not different variables (e.g. original unfiltered signals, filtered signals, or trigger lines). For example, Figure 11 shows the raw signal of the vibration at tip of the hitting device (purple line) and the filtered signal recorded by the SLV (red line).

### Generation of the tomographic image

To generate the tomographic image, algebraic reconstruction techniques will be used, namely the Simultaneous Iterative Reconstructive Technique (SIRT) [ Kaks & Slaney, 2001]. These methods firstly require to specify the sonic ray paths, i.e. transmitter and receiver positions, and will assume that the transmission is straight between both points. Note that due to the high heterogeneity of the inspected medium the wave will suffer significant refraction and diffraction effects. Therefore, the previous assumption of a straight ray path is far from reality. However, given the great difficulty in predicting the real ray path, this is a necessary simplification that has led to good results in the past [ Luchin *et al*., 2018; Zielinska & Rucka, 2018].

**Figure 12.**
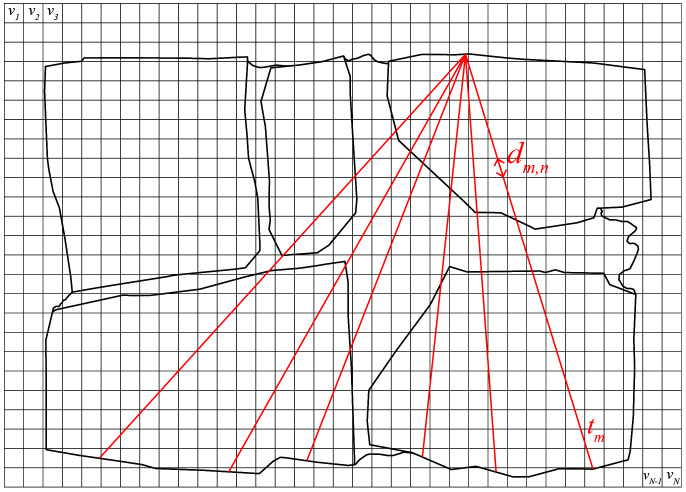
Schematic diagram of sonic tomography with cross-section discretized into pixels (gray grid) and example of sonic rays considered between emission and reception points (red lines).

The application of algebraic techniques are based on the estimation of the velocities spatial distribution over a cross-section, discretizing it into a square grid of *N* cells. As an example, Figure 12 shows the typical grid assumed for one of the walls cross-sections. The overall dimensions of the walls cross-section, which will be explained later in detail, led to a rectangular grid of 35 x 25 cells, which will be the pixels of the tomographic image, for a total of *N* = 875. Typically, 7 emission and 7 reception positions were defined per cross-section, located in opposite faces of the walls. Interpolated sonic rays were assumed between reception points, leading to an average total of 112 rays used to generate the tomographic reconstruction of the cross-section.

The travel time of the ray path going through several cells of the discretized cross-section can be written as:


$$t_{m}=\sum_{n=1}^{N}d_{m,n}/v_{n}\;(1)$$

where *t _m_* is the travel time of the *m*th ray, 1 < *m* < *M = 112*, *v*_*n*_ is the velocity associated with the *n*th cell, 1 < *n* < *N = 875*, and *d _m,_*_*n*_ is the transition distance of the *m*th ray through the *n*th cell. Note that most of *d _m,n_* coefficients are zero because the path does not pass through most pixels. This process results of a system of *M* linear equations of *N* variables that can be expressed in matrix form:


t=Ds(2)

where *t* = [ *t*_1_, *t*_2_,…, *t _M_*] *^T^* is the vector of measured times, *D* is the 35 x 25 matrix with the values of the distances covered by the *m*th ray through the *n*th pixel, and *s* = [ *s*_1_, *s*_2_,…, *s _N_*] *^T^* is the vector that contains the discretized values of the slowness at pixel *n*, whose inverse is the velocity *v _n_* = 1/ *s _n_*. The matrices *t* and *D* are known. Thus, the tomographic reconstruction consists in determining the vector *s*: 


$$\;s=D^{-1}t\;(3)$$

**Figure 13.**
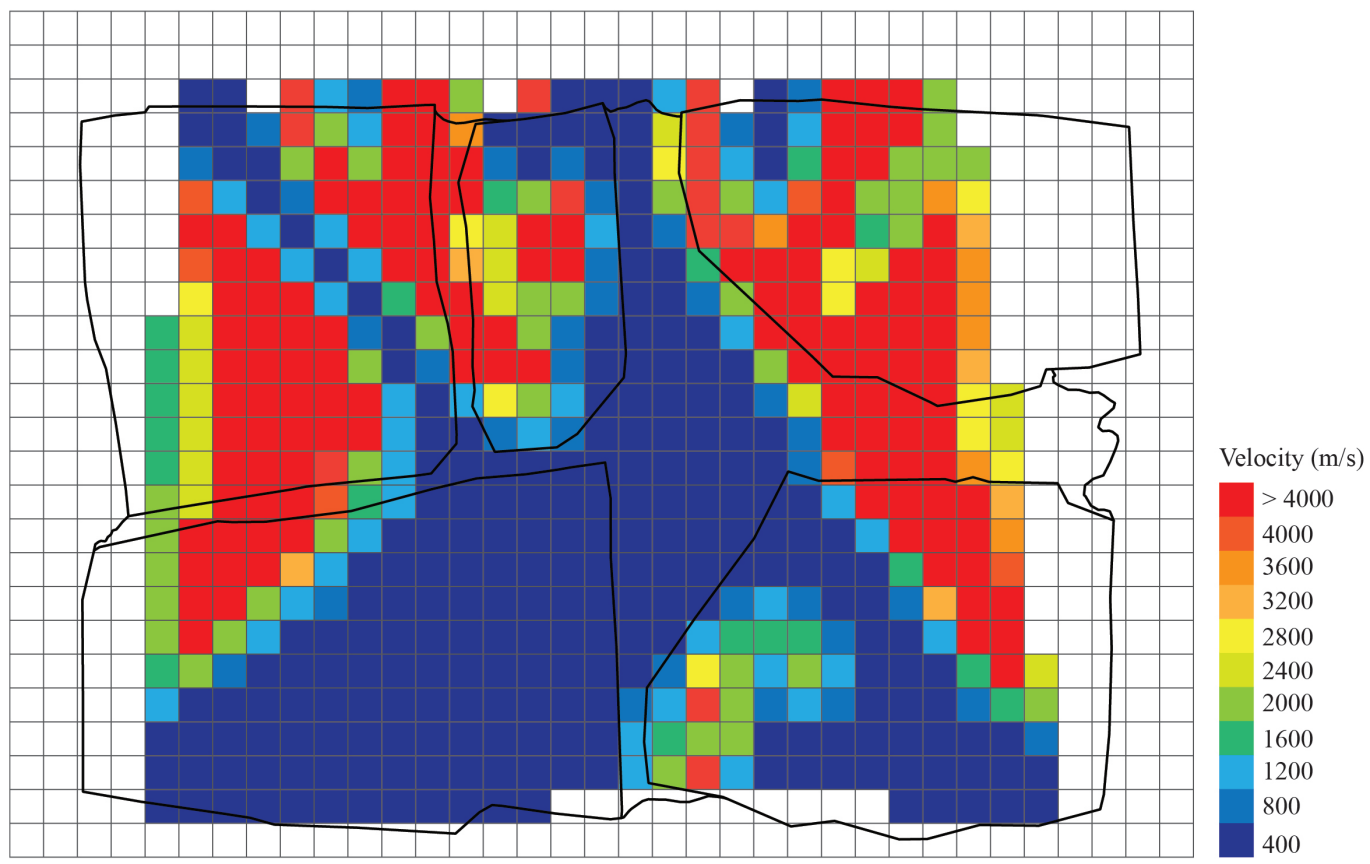
Example of tomographic reconstruction image of a wall cross-section.

To solve the resulting system of equations, iterative methods (e.g. SIRT) are required. The first iteration, *s _n_^(0)^*, consists of assigning the same slowness to all pixels (equal to the inverse of the average velocity value obtained from all measurements at the cross-section). The iteration process continues until finding convergence and the travel time obtained matches the measured one within an established error. Each iteration, *k*, the new solution, *s _n_^(k)^*, is computed by correcting the previous solution, *s _n_^(k-1)^*, with the following expression: 


$$S_{n}^{\left(k\right)}=S_{n}^{\left(k-1\right)}+\frac{t_{m}-t_{m}^{\left(k-1\right)}}{\sum_{n=1}^{N}{d^{2}}_{m,n}}d_{m,n}\;(4)$$

where *t _m_^(k-1)^* is the computed travel time of the *m*th ray based on the *(k-1)*th solution. The correction applied to the slowness of *n*th cell is thus obtained by calculating the difference between the measured travel time and the computed one, normalized by the squared sum of the distances that the *m*th ray traveled through each pixel. This correction value is assigned to each pixel crossed by the *m*th ray, weighted by the corresponding distance traveled through that pixel, *d _m,n_*. The process ends with a tomographic image that shows the velocity spatial distribution across the cross-section ( Figure 13). Smoothing filters can be later applied for an easier visualization of the results. Note also that the process of generating the tomographic image can be also done in 3D assuming voxels in terms pixels for its calculation, but following the same process previously described.

## Results

Six stone masonry walls, representative of historical typologies with different interior geometries, were constructed at the laboratory by a professional stonemason. Three walls were constructed following a representative irregular masonry morphology and the other three were constructed according to a typical ashlar masonry typology. The construction of the walls was carefully documented, including the generation of detailed photogrammetric models of each single stone. Tomographic inspections using the developed automated system were carried out in the six walls and the data was processed following the process previously described and using the automatic processing tools developed. The objective of the walls is twofold: (a) test the capabilities of the developed automated sonic tomography systems and methods for the inspection of historical masonry walls; and (b) assess the possibilities of tomographic inspections to provide detailed information of the interior of structural elements and distinguish different stone masonry construction typologies.

### Stone masonry walls

**Figure 14.**
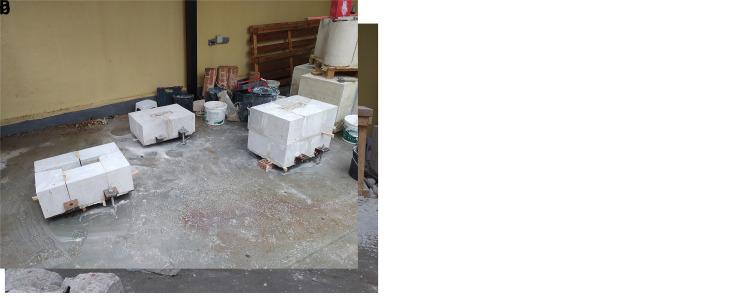
Construction of: ( **a**) irregular Stone masonry walls; and ( **b**) ashlar masonry walls.

The walls were constructed at the ITEFI laboratory ( Figure 14). The dimension of the walls was 60 x 40 x 90 cm (length x width x height) in the case of the irregular stone masonry walls, and 70 x 50 x 80 cm in the case of the ashlar masonry walls. The stone used in all walls is a *Campaspero* limestone, which is intrasparite type of limestone from the north west of Spain with a reported average compressive strength of 136 MPa ( *σ =* 12 MPa). The mortar used is a low strength mortar, mix of lime putty mortar from Morón (traditional lime products producers) and fine aggregates, namely sand, marble powder. Compressive strength tests performed on mortar specimens casted during the construction process resulted in an average value of 2.1 MPa ( *σ =* 0.48 MPa).

**Figure 15.**
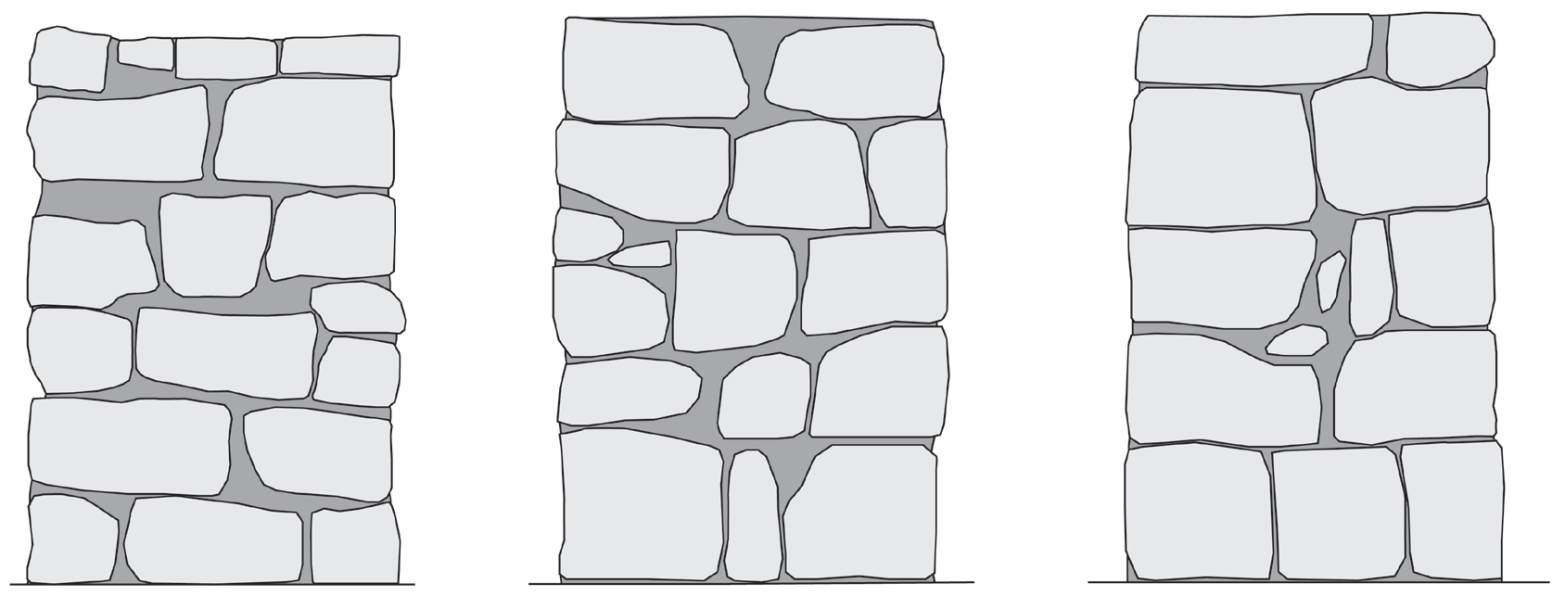
Elevations of the three irregular stone masonry walls.

The irregular masonry walls are composed of 5-6 rough horizontal courses of varying height ( Figure 15). At the same time, the courses are composed of non-parallelepiped stones of different sizes. The walls are roughly composed of two leaves with an inner core of small dimensions. Through-stones covering the whole width of the wall are placed at some courses in all three walls.

**Figure 16.**
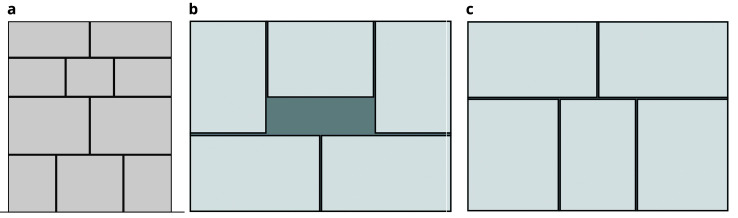
( **a**) Elevations of the three ashlar stone masonry walls; and ( **b**) bottom cross-sections; ( **c**) upper cross-sections.

The ashlar stone masonry walls are composed of 5 horizontal courses of varying height ( Figure 16a), ranging from 24 cm (bottom courses) and 15 cm (upper courses). The two bottom cross-sections have the same cross-section, but mirrored, composed of two leaves with an inner core ( Figure 16b). The two upper cross-sections shared also the same morphology (but mirrored) and have no inner core ( Figure 16c).

### Walls photogrammetry and 3D models

The construction of the walls was carefully documented, including the generation of detailed photogrammetric models of each single stone before they were placed. As a result, accurate 3D models of the walls were prepared that include their inner configuration. The workflow for the generation of the geometrical models of the masonry walls is based on the framework proposed by [ Pantoja-Rosero *et al*., 2023] to generate geometrical digital replicas of stone masonry elements.

The first step is to generate the photogrammetric models of each stone using a Structure-from-Motion (SfM) technique, which reconstruct an object in 3D based on multiple overlapped images of the object taken from different locations at different angles. This step was not straightforward because the stonemason could not use the stones as they were when they arrived from the quarry. The stones needed to be appropriately cut for them to fit well geometrically and compose the walls with the specific dimensions and roughly respect the horizontal courses and vertical planes of the four sides. Thus, before the stonemason mortared the stones within the wall, he laid each course dry ( Figure 14a) and we numbered the stones. After surveying each stone, they were returned back to the dry course and the stonemason was able to place and mortar it within the wall.

**Figure 17.**
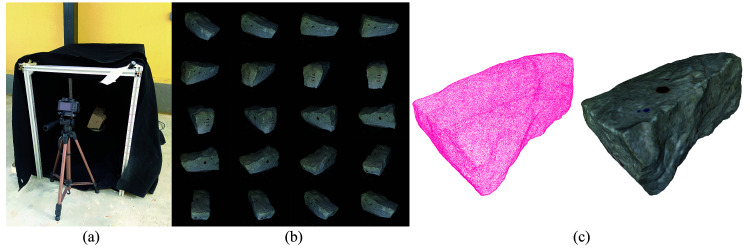
Workflow for the generation of the SfM models of the individual stones: ( **a**) light box for capturing the images of each stone; ( **b**) multiple images of the stone captured from the light box; and ( **c**) resulting SfM 3D models of the stone.

The system used to collect the images necessary to generate the SfM models was a light box made at the laboratory and a photographic camera ( Figure 17a). This system was devised to reduce the time necessary to capture the images. Since multiple overlapping images of each stone from all angles are needed, note that between 100 and 150 images were typically used for the reconstruction of one stone, the process can become time consuming and needed to be automated. The light box is composed of an aluminum frame, a thick dark fabric, a rotating platform and lights. The rotating platform was activated by a motor and the velocity and rotation direction could be controlled. A camera is placed on a tripod at the uncovered end of the box and photos are taken automatically while the stone placed at the platform rotates. After two full rotations, the stones needed to be manually rotated on the platform so that images are taken from all the sides ( Figure 17b). In the end, a detailed 3D reconstruction of each stone is generated ( Figure 17c).

**Figure 18.**
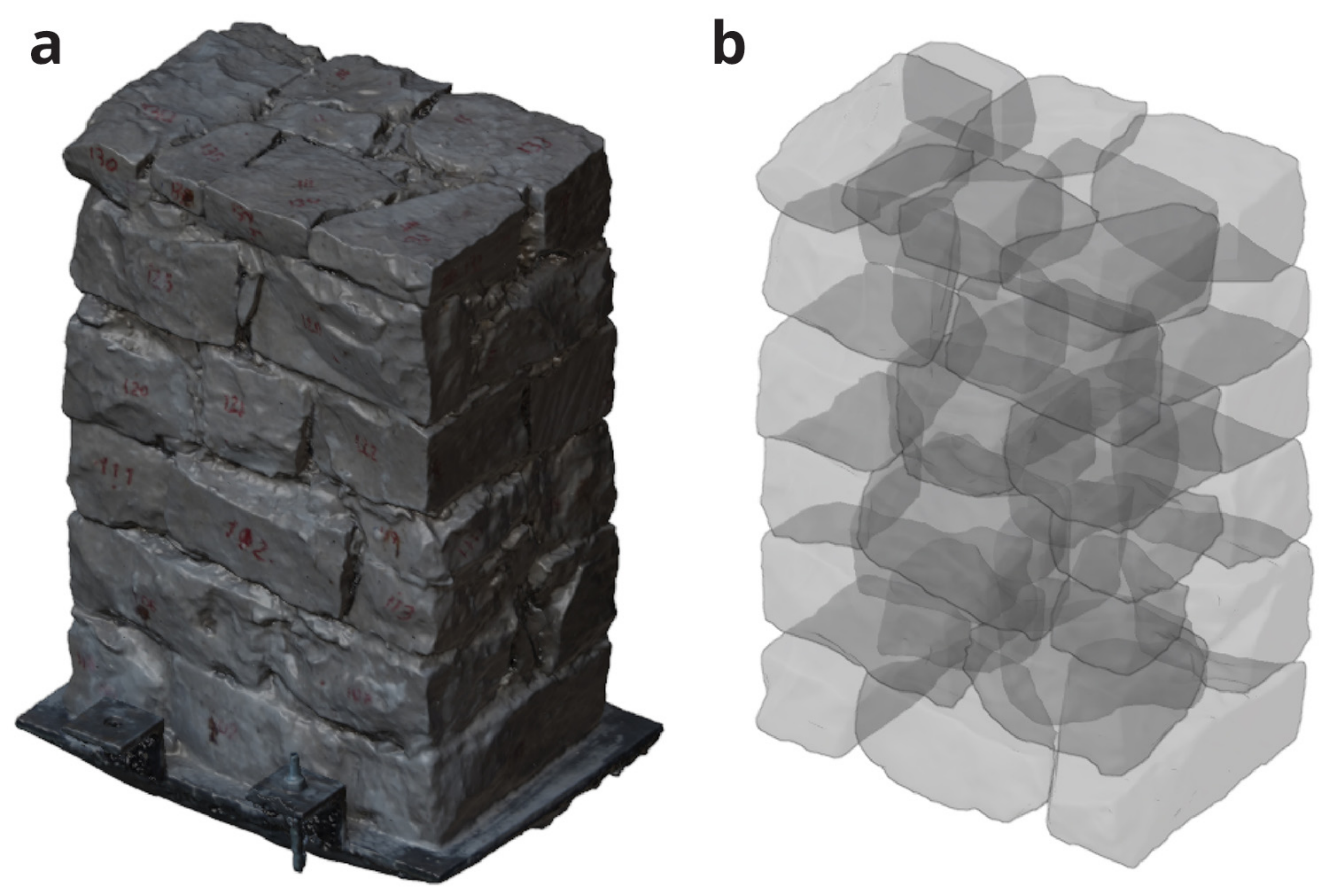
( **a**) SfM model of one of the irregular masonry walls; and ( **b**) complete 3D reconstruction of the stone masonry wall including all individual stones (x-ray view).

After the photogrammetric survey of all stones, a SfM model of the constructed wall is also carried out ( Figure 18a). Once all the models are ready (wall and stones), all models were aligned to obtain a full 3D digital model of each stone masonry wall that includes the position of all stones and thus provides information about its inner condition ( Figure 18b). The alignment process is carried out using the align tool from the open source software MeshLab [ Cignoni *et al*., 2008]. Such tool allows to align point clouds based on matching points, e.g. the ink marks of the stones, which were visible in both wall and individual stone models, or sufficiently distinctive features of the individual stones.

This process was only followed for the irregular stone masonry walls (Walls 1–3). The ashlar stones were already precisely cut at the quarry with the specific dimensions shown in Figure 16. The construction of the ashlar walls (Walls 4–6) was simpler, and individual SfM models of each ashlar were not deemed necessary. The interior of the walls is known and only general SfM model of the whole constructed walls were carried out.

As a result of the photogrammetric survey process, detailed information of the interior of the six walls was obtained. This information was essential for to evaluate the capabilities of the tomographic inspection to distinguish different stone masonry construction typologies. The SfM models serve as ground-truth models to compare with the tomographic images. All six models (walls and stones) are publicly available in [ Ortega *et al*., 2023a].

Finally, simultaneous to the photogrammetric surveys, ultrasonic measurements were carried out for each stone and mortar specimens to characterize their specific wave propagation characteristics, namely longitudinal and transversal waves velocity. The stones were also weighted and, since the exact volume of the stones is known, the densities could be calculated. As a result, the dynamic modulus of elasticity ( *E*) and Poisson’s ratio ( *ν*) of each stone could also be computed. Table 2 and Table 3 show the average results of the ultrasonic measurements carried out in the stones and mortar specimens. Overall, the stones reveal a high homogeneity with low coefficient of variation in terms of density and ultrasonic wave velocities (both longitudinal and transversal). Variations in terms of modulus of elasticity are higher but still below 20% and average values are similar in all walls. The lime mortar shows higher variability between the walls, particularly in terms of dynamic modulus of elasticity and compressive strength. In the case of the ashlar stone masonry walls, a mortar of lower mechanical characteristics was applied for the joints, since their main role is to receive the ashlars.

**Table 2.** Average results of ultrasonic tests carried out in the stones (in brackets coefficient of variation).

Wall	*ρ* (kg/m ^3^)	*V _L_* (m/s)	*V _T_* (m/s)	*ν*	*E* (GPa)
Wall 1	2379 (4%)	4344 (8%)	2641 (7%)	0.20 (14%)	40 (18%)
Wall 2	2397 (4%)	4347 (8%)	2645 (9%)	0.21 (16%)	41 (19%)
Wall 3	2403 (3%)	4260 (6%)	2579 (6%)	0.21 (6%)	39 (14%)
Wall 4-6	2420 (5%)	4283 (5%)	2606 (5%)	0.21 (4%)	40 (14%)

**Table 3.** Average results of ultrasonic tests carried out in the mortar specimens (in brackets coefficient of variation).

Wall	*ρ* (kg/m ^3^)	*V _L_* (m/s)	*V _T_* (m/s)	*ν*	*E* (GPa)	*f _c_* (MPa)
Wall 1	1747 (1%)	2116 (0%)	1324 (5%)	0.17 (30%)	7.2 (4%)	2.39 (20%)
Wall 3	1775 (1%)	1941 (10%)	1129 (1%)	0.23 (32%)	5.6 (8%)	1.82 (18%)
Wall 4-6	1750 (-)	1264 (1%)	820 (0%)	0.14 (8%)	2.7 (1%)	0.81 (0%)

### Sonic tomography results

**Figure 19.**
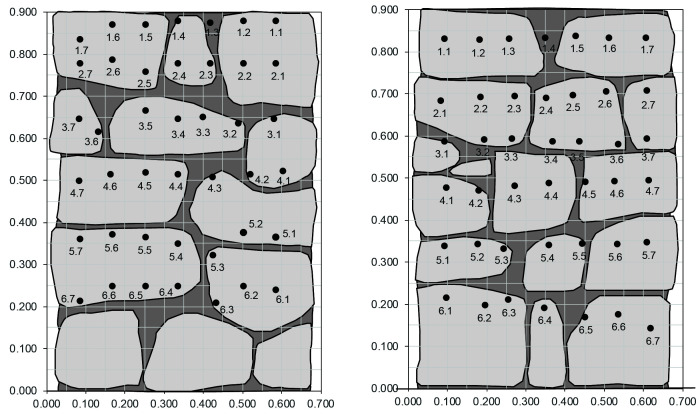
Example of layout of emission (left) and reception (right) points for the tomographic inspection of the irregular masonry walls, located on opposite surfaces of the wall. Dimensions in meters.

**Figure 20.**
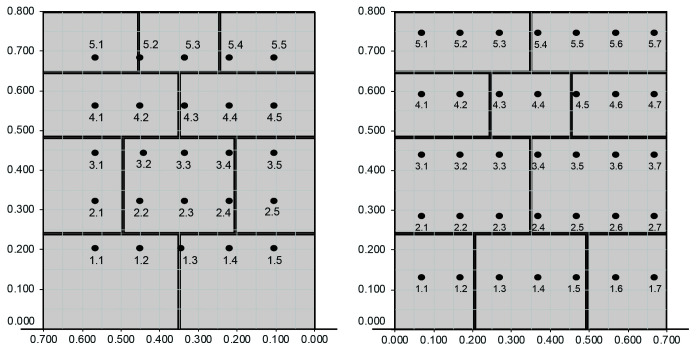
Example of layout of emission (left) and reception (right) points for the tomographic inspection of the ashlar masonry walls, located on opposite surfaces of the wall. Dimensions in meters.

The tomographic image will be generated using the transmission method, i.e. the emission and reception are located at opposite sides of the walls and thus the sonic ray travels through the masonry. For each wall, the location of emission and reception points varied slightly, adapted to the morphology and texture of the wall surface. In the case of the regular walls, the location was kept basically the same for all three. In general, the tests tried to follow a grid, which had to be adapted to the location of the stones, favoring emission and reception points located on the stones instead of mortar joints. Figure 19 shows an example of the layout of emission and reception used for the inspection of one of the irregular walls (Walls 1–3), which follows roughly a 6 x 7 (rows x column) grid for emission and reception. Figure 20 shows the regular emission grid of 5 x 5 (rows x column) and reception grid of 5 x 7 (rows x column), followed for the inspection of the ashlar masonry walls (Walls 4–6). 

**Figure 21.**
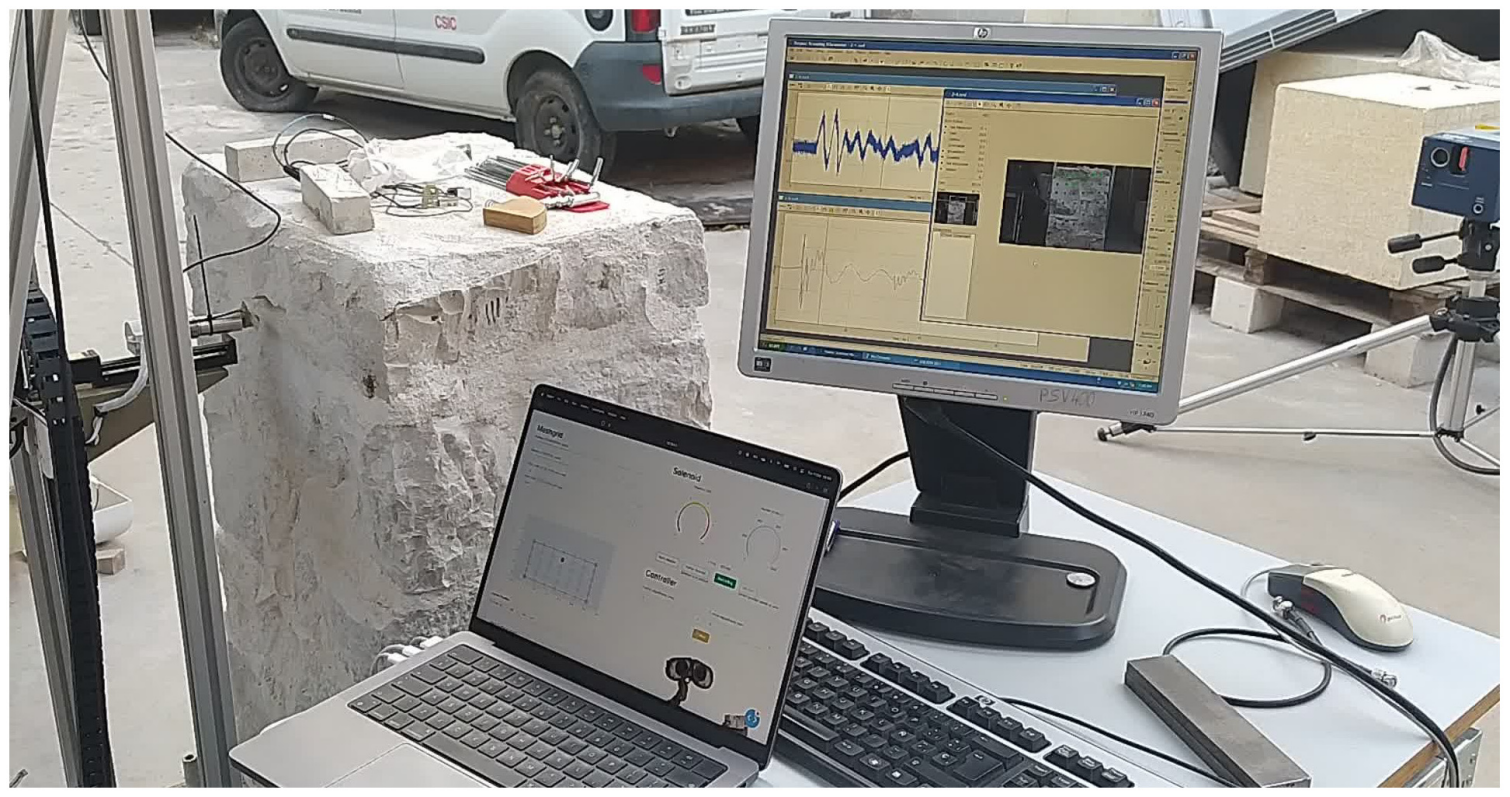
Tomographic inspection test setup. From left to right: AHS hitting at the emission points on the wall surface, computer controlling the AHS with the software developed, data acquisition system of the SLV showing the reception of the signal in real time and SLV measuring the vibrations at the reception points on the opposite surface of the wall.

**Figure 22.**
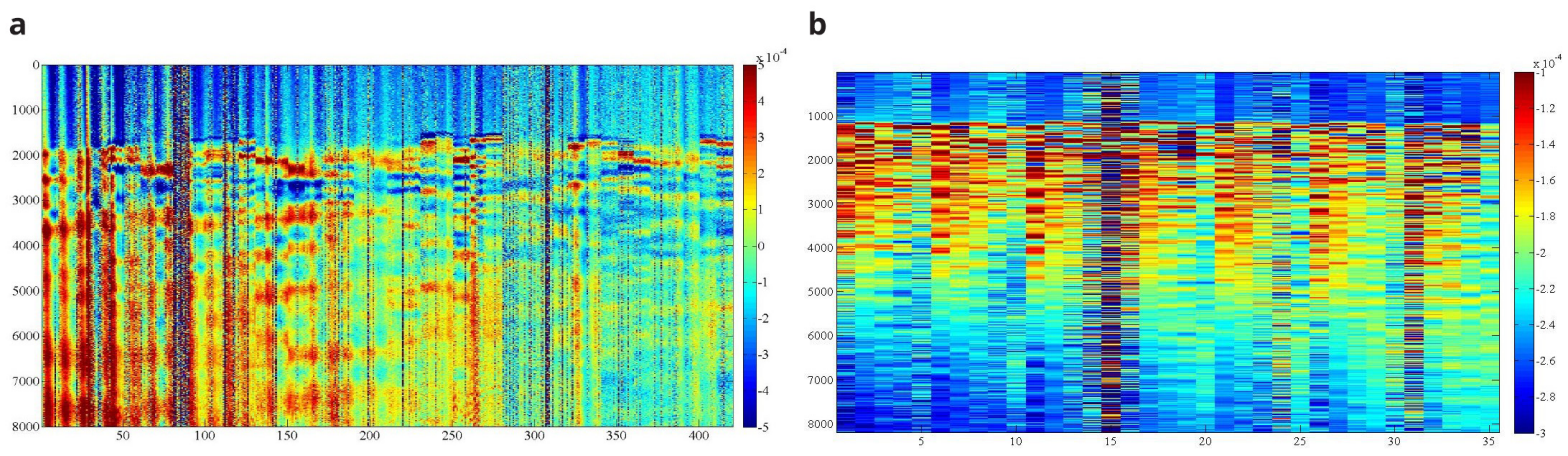
Example of B-scan image obtained for one emitting point at the irregular ( **a**) and ashlar ( **b**) masonry wall. X axis shows number of measurements, Y axis shows number of samples per measurement and Z (colormap) shows signal amplitude in terms of voltage (V).

Figure 21 shows an image of the test setup with the AHS located in one side of the wall, while the SLV measures the vibrations on the opposite surface. For each emission point, vibration measurements at all reception points were recorded. This led to a total of approximately 420 measurements per inspection point in the case of the irregular walls and 350 measurements in the case of the regular walls (due to the 10 hits performed per reception point for later averaging). Figure 22 shows the received raw signal (without processing) obtained from a single emission point for the irregular (a) and ashlar (b) masonry walls. The hitting frequency of the AHS is around 3 hits/s. Therefore, the inspection time of the whole wall was around 1.5–2 hours (around 3 min per emission point). Over 17,000 measurements were collected for the irregular walls. In the case of the ashlar masonry walls, the data was averaged automatically during the collection by the SLV data acquisition system. That is why Figure 22b shows only 35 measurements per inspection point. The raw data collected per wall is also publicly available in [ Ortega *et al*., 2023b].

**Figure 23.**
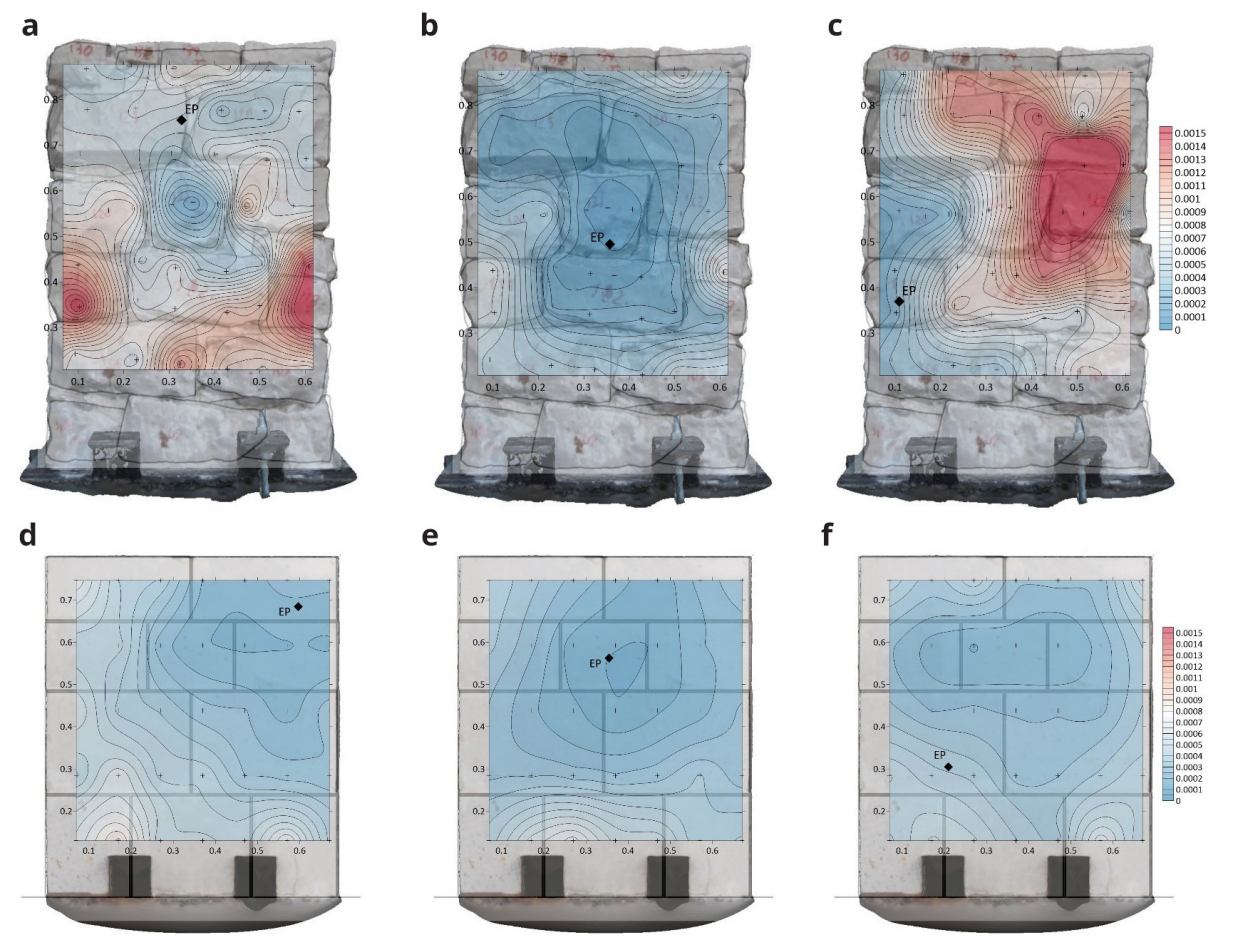
Sonic transmission contour maps for wall 1 ( **a**– **c**) and wall 4 ( **d**– **f**) in terms of travel time (in seconds). The location of the emission point for each contour map is marked with the letters EP.

The results of the inspections are firstly presented in terms of sonic transmission maps. Figure 23 shows the results in terms of travel time (in seconds) for Wall 1 and 4 for different emission points, marked in the figure with the words EP. The difference between the irregular and ashlar masonry walls is evident in the maps. Travel times in the irregular masonry walls reach high values in locations that are far from the emission point, which illustrates that there is no direct travel path between emission and some reception points (probably due to the presence of voids and discontinuities). In the case of the ashlar masonry walls, the travel time increases progressively when the distance between emission and reception point increases, indicating a more homogeneous material. 

**Figure 24.**
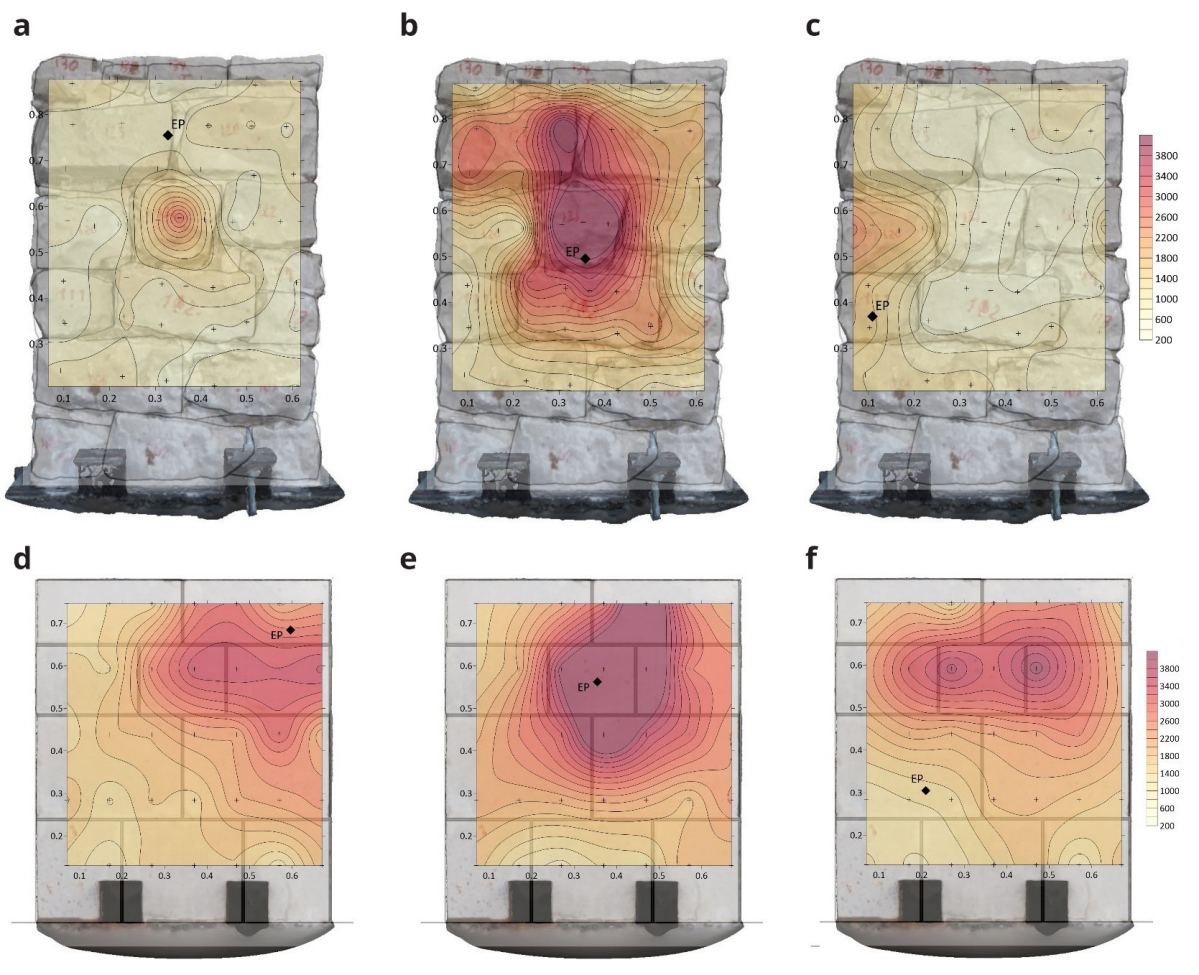
Sonic velocity contour maps for wall 1 ( **a**– **c**) and wall 4 ( **d**– **f**) in terms of velocity (in m/s). The location of the emission point for each contour map is marked with the letters EP.

Such conclusions are more evident when looking at the results in terms of velocities. Figure 24 shows the results in terms of velocity contour maps for the same walls same emission points from Figure 23. In average, the velocities obtained for the irregular masonry wall, approx. 1000 m/s, are far lower than the ones for the ashlar masonry wall, approx. 2000 m/s. The velocities are, nevertheless, rather homogeneous throughout the irregular masonry wall. The higher velocities obtained at the mid-center of the wall (e.g., Figure 24b) are due to the presence of a through stone (stone that goes through the whole thickness of the wall). The presence of such stone can be clearly detected from the results and has an essential influence of the acoustic transmission throughout the wall. In the case of the ashlar masonry wall, the results show clearly that the velocities at the upper part of the wall, which is solid and has no inner core ( Figure 16), are higher. The results thus illustrate the sensitivity of the sonic transmission to the presence of an inner core in the wall.

**Figure 25.**
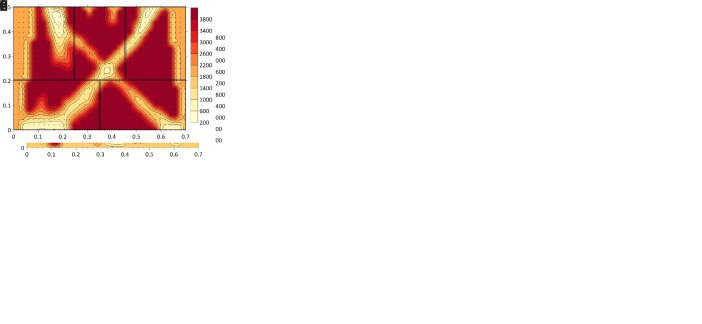
Tomographic images at approximately 0.5-0.6 m height from: ( **a**) Wall 1 (h = 0.53 m); ( **b**) Wall 2 (h = 0.49m); ( **c**) Wall 3 (h = 0.55 m); ( **d**) Wall 4 (h = 0.57 m); ( **e**) Wall 5 (h = 0.60 m); ( **f**) Wall 6 (h = 0.56 m).

For each inspected wall, several tomographic slices could be done at different heights. Figure 25 shows the tomographic images of the cross-sections of all six walls at a height of 0.5-0.6 m, for illustrative purposes. The images show the computed velocity distribution at each cross-section. Additionally, the real cross-sections of the walls extracted from the complete SfM models is overlapped on the tomographic image, which allows to compare the maps with the ground-truth and facilitates the interpretation of the results.

The highest values of velocity (closer to the sonic velocity through the stone, i.e. around 4,000 m/s) indicate a good transmission whereas the lowest values probably indicated the presence of voids or cracks in the sonic ray path. Even though the interpretation of the results is not straightforward, the results show the sensitivity of the tomographic reconstruction to the variations in the inner morphology of the masonry walls. This is particularly evident when comparing the ashlar masonry walls with the irregular masonry walls. In the case of the ashlar masonry walls, the images show rather homogeneous maps with high velocities, close to the one from the stone. Additionally, the three cross-sections lead to similar tomographic reconstructions and little variations can be observed among the walls. In the case of the irregular masonry walls, the three cross-sections are significantly different. Large areas with low velocities show the poor connectivity among some inner parts. For example, the tomographic image of wall 2 ( Figure 25b) shows a large central area with low velocities, which matches well the real cross-section of the wall with no through stones. Conversely, the tomographic image of wall 1 shows high velocities in the center, which correspond to the presence of the through stone at that location.

These preliminary results evidence the difficulty of the interpretation of the results for heterogeneous and discontinuous materials that are common in historic structures. The correlation between the tomographic images and inner morphology of masonry construction is not evident, but results show that the acoustic wave propagation attributes is clearly sensitive to the cross-section characteristics. The results open new paths for investigation. For example, new processing algorithms based on deep learning could be applied to process the tomographic images and optimize the interpretation of the data, aiming to provide accurate information about the internal geometry of the inspected structure. Nevertheless, to train such algorithms and understand better the correlation between acoustic velocities and inner geometry, a great amount of data is needed. For that purpose, the flexible automated tomographic inspection system that has been designed in the present research is essential, which allows collecting a large amount of data in little time.

## Conclusions

The paper has shown the development of a novel automated sonic tomography system designed by the authors and particularly adapted to inspect masonry walls of heritage structures. The system is composed of a new Automatic Hitting System, including a mechanical and an electronic system, that generates the acoustic wave and a Scanning Laser Vibrometer to receive the signal. The system is designed mainly aiming to overcome common limitations of existing techniques by automating the tomographic inspection. Conventional methods are extremely time consuming because the inspections are carried out manually. That is why their use is limited in practice and focus on local inspections of a few elements.

The system was tested and validated for the inspection of six small scale walls constructed at the laboratory and simulating representative stone masonry construction typologies of heritage structures. Mainly two types of walls were constructed: irregular stone masonry walls and ashlar masonry walls. A full inspection of the wall could be done in less than two hours, but a 2D inspection of one cross-section of the wall could be performed in less than 10 minutes. The novel system can collect thousands of data per day of a structural element, which also allows to build 3D tomographic images of entire elements. The automation was also implemented at the level of processing the data by developing new software, which is also essential given the great amount of data generated.

During the construction of the walls, a thorough documenting workflow was defined aiming to collect geometric and material information of the individual stones before they were placed. Such approach allowed for a comprehensive understanding of the walls and its constituents, including their geometric configuration within the wall. Thus, the resulting tomographic images could be compared with the ground-truth. The results obtained show that the system is able to provide relevant information of the internal morphology of the inspected walls. The tomographic inspection is sensitive to slight variations in the inner morphology, e.g. among the different irregular masonry walls, but also to significant variations, e.g. between ashlar and irregular masonry walls.

The outcomes of the present research are expected to highlight the potential of the tomographic technique to obtain quantitative information about the interior of heritage structures. This is essential for conservation purposes and for making decisions on how to intervene. However, for the tomography technique to become an efficient on-site non-destructive evaluation tools for practitioners a more practical implementation is required, at the operational, processing and interpretation level. The paper is expected to be a step forward in this direction, while opening new lines of research that still need to be tackled. With that purpose, the data, software and models generated during the present research have been made publicly available in open-source repositories.

## Data Availability

Zenodo. DocumeNDT stone masonry walls geometry: Photogrammetric models dataset DOI: 10.5281/zenodo.7713700 This repository contains detailed geometric data from the six stone masonry walls constructed for the experimental campaign. It is structured in 2 levels of folders: At first level, the 6 folders correspond to the 6 tested stone masonry walls (Wall 1-6). At second level, for each wall, there are two folders. The first folder contains the photogrammetric model of each wall, e.g., "W1" is the photogrammetric model of Wall 1, and a photograph showing the same wall. The second folders contains individual photogrammetric models of each stone composing the walls and their exact location within the walls, e.g., "Stone 101". Zenodo. DocumeNDT stone masonry walls tomographic inspection dataset [Data set]. Zenodo. DOI: 10.5281/zenodo.7713744 This repository contains data from the sonic tomography inspections of the six stone masonry walls constructed for the experimental campaign. It is structured in 2 levels of folders: At first level, the 6 folders correspond to the 6 tested stone masonry walls (Wall 1-6). At second level, for each wall, there are three folders: The folder 'Coordinates' contains: (1) the coordinates of the emission and reception points; (2) diagrams of the emission and reception locations in elevation; and (3) readme file with specific details about the inspection, e.g., number of emission and reception points The folder 'Emission raw signal' contains the recorded emission signal for each emission location The folder 'Reception raw signal' contains the recorded reception signal for each emission location Data are available under the terms of the Creative Commons Attribution 4.0 International license (CC-BY 4.0).

## Software availability

- Meersman, M.L.F. (2023a) DocumeNDT Controller (v1.0.0) [Software]. Zenodo. DOI: 10.5281/zenodo.7778059.
- Meersman, M.L.F. (2023b) DocumeNDT Data Processing (v1.0.0) [Software]. Zenodo. DOI: 10.5281/zenodo.7778105.

Data are available under the terms of the Creative Commons Attribution 4.0 International license (CC-BY 4.0).
