# Supplementary material for: An automated sonic tomography system for the inspection of historical masonry walls
Source: Open Res Eur. 2023 Apr 21;3:60. [Version 1] doi: 10.12688/openreseurope.15769.1 (PMC10445949; doi:10.12688/openreseurope.15769.1)
Supplement: Peer review history

# Peer review: Reviewer response for version 1

**DOI:** 10.21956/openreseurope.17034.r31444

**Published:** 2023-05-22

**Authors:** Jorge Gosálbez Castillo

**Affiliations:** 1 Instituto de Telecomunicaciones y Aplicaciones Multimedia (ITeAM), Universitat Politecnica de Valencia, Valencia, Valencian Community, Spain

The aim of this work is to reconstruct the inner of historical masonry walls. The paper focuses on the developing of a tomographic system based on multiple spatial impact signals and multiple spatial reception signals combined with signal processing algorithms. An algebraic reconstruction algorithm (SIRT) is used to estimate the velocity map of the wall.

 For this aim, a XYZ frame is implemented to allow to position the automatic hammer. This module is composed by a coarse XY positioner to place the hammer over the predefined meshing on the wall and a fine Z positioner to approach the appropriate distance and bridge the typical irregularities of the masonry walls.

 The reception stage is composed by an automatic laser that is able to automatically point to different spatial position (3D rotating head), but it is not capable to point simultaneously, therefore it requires periodic impacts. In addition, signal processing algorithms are required to improve the SNR of the signals and properly detect the time of flight.

 The entire system is controlled by a software and the excitation and reception stages are synchronize by an accelerometer attached to the tip of the hammer.

 The tomographic reconstruction algorithm is based on the SIRT method (algebraic method) which is fed with the time of flight and the sparse matrix of the distance contribution of each pixel to each ray. The slowness is obtained from the SIRT method and velocity is obtained as the inverse of the slowness. Although this algorithm assumes straight line propagation, the obtained results support this restriction.

 Finally, the authors present several results of the reconstruction of different walls. It is important to highlight the digital photogrammetry that supports author’s arguments.

 The paper is well organized and structured and easy to read.

 In this reviewer’s opinion, the paper can be indexed in its present form.

Is the rationale for developing the new method (or application) clearly explained?

Yes

Is the description of the method technically sound?

Yes

Are the conclusions about the method and its performance adequately supported by the findings presented in the article?

Yes

If any results are presented, are all the source data underlying the results available to ensure full reproducibility?

Yes

Are sufficient details provided to allow replication of the method development and its use by others?

Yes

Reviewer Expertise:

Ultrasounds, signal processing, non-destructive testing,

I confirm that I have read this submission and believe that I have an appropriate level of expertise to confirm that it is of an acceptable scientific standard.

# Peer review: Reviewer response for version 1

**DOI:** 10.21956/openreseurope.17034.r31240

**Published:** 2023-05-05

**Authors:** Marco Funari

**Affiliations:** 1 School of Sustainability, Civil and Environmental Engineering, University of Surrey, Guildford, England, UK

This paper presents a new automatic sonic tomography system for the inspection of historical masonry walls.

 In my understanding, the main novelty consists of the design of a frame which allowed the translation of the equipment along X Y, and Z. Apart from that, the paper presents an interesting laboratory application of the apparatus, and the authors also made some extra effort in order to apply additional techniques such as digital photogrammetry in order to foster the arguments of their research.

 Overall the paper looks well-designed, and the Figures and Tables are clear.

 The comparison between irregular masonry walls is of particular interest, attracting interest for future applications of the proposed methodology.

  In this reviewer’s opinion, the paper can be indexed in its present form.

Is the rationale for developing the new method (or application) clearly explained?

Yes

Is the description of the method technically sound?

Yes

Are the conclusions about the method and its performance adequately supported by the findings presented in the article?

Yes

If any results are presented, are all the source data underlying the results available to ensure full reproducibility?

Yes

Are sufficient details provided to allow replication of the method development and its use by others?

Yes

Reviewer Expertise:

Historic Masonry Structures, Structural assessment and NDT

I confirm that I have read this submission and believe that I have an appropriate level of expertise to confirm that it is of an acceptable scientific standard.
